# miR-125b Acts as a Tumor Suppressor in Breast Tumorigenesis via Its Novel Direct Targets ENPEP, CK2-α, CCNJ, and MEGF9

**DOI:** 10.1371/journal.pone.0076247

**Published:** 2013-10-03

**Authors:** Andrea Feliciano, Josep Castellvi, Ana Artero-Castro, Jose A. Leal, Cleofé Romagosa, Javier Hernández-Losa, Vicente Peg, Angels Fabra, Francisco Vidal, Hiroshi Kondoh, Santiago Ramón y Cajal, Matilde E. LLeonart

**Affiliations:** 1 Oncology and Molecular Pathology Group, Pathology Department, Institut de Recerca Hospital Vall d’Hebron, Barcelona, Spain; 2 Institut d’Investigació Biomèdica de Bellvitge (IDIBELL), Barcelona, Spain; 3 Banc de Sang i Teixits, Barcelona, Spain; 4 Department of Geriatric Medicine, Graduate School of Medicine, Kyoto University, Kyoto, Japan; IPMC, CNRS UMR 7275 UNS, France

## Abstract

MicroRNAs (miRNAs) play important roles in diverse biological processes and are emerging as key regulators of tumorigenesis and tumor progression. To explore the dysregulation of miRNAs in breast cancer, a genome-wide expression profiling of 939 miRNAs was performed in 50 breast cancer patients. A total of 35 miRNAs were aberrantly expressed between breast cancer tissue and adjacent normal breast tissue and several novel miRNAs were identified as potential oncogenes or tumor suppressor miRNAs in breast tumorigenesis. miR-125b exhibited the largest decrease in expression. Enforced miR-125b expression in mammary cells decreased cell proliferation by inducing G2/M cell cycle arrest and reduced anchorage-independent cell growth of cells of mammary origin. miR-125b was found to perform its tumor suppressor function via the direct targeting of the 3’-UTRs of ENPEP, CK2-α, CCNJ, and MEGF9 mRNAs. Silencing these miR-125b targets mimicked the biological effects of miR-125b overexpression, confirming that they are modulated by miR-125b. Analysis of ENPEP, CK2-α, CCNJ, and MEGF9 protein expression in breast cancer patients revealed that they were overexpressed in 56%, 40–56%, 20%, and 32% of the tumors, respectively. The expression of ENPEP and CK2-α was inversely correlated with miR-125b expression in breast tumors, indicating the relevance of these potential oncogenic proteins in breast cancer patients. Our results support a prognostic role for CK2-α, whose expression may help clinicians predict breast tumor aggressiveness. In particular, our results show that restoration of miR-125b expression or knockdown of ENPEP, CK2-α, CCNJ, or MEGF9 may provide novel approaches for the treatment of breast cancer.

## Introduction

The incidence of malignancy is increasing worldwide, to such an extent that cancer has replaced heart disease as the leading cause of disease-related mortality [1]. Breast cancer is the second leading cause of cancer-related deaths in the USA and Europe. Mortality from this disease remains high because current therapies are limited by the emergence of therapy-resistant cells [2].

miRNAs are small (18- to 25-nucleotide-long) single-stranded noncoding RNAs that regulate gene expression at the posttranscriptional level by binding to the 3'-UTR of the target messenger RNA (mRNA), thereby causing translational repression or degradation. However, some miRNAs have been shown to bind to the open reading frame, the 5’-UTR, or the promoter of the target mRNA to cause downregulation or upregulation of gene expression [3-7]. Thus, miRNAs are considered key regulators of gene expression at transcriptional and posttranscriptional levels. In addition, miRNAs play essential roles in the regulation of biological processes, including cell proliferation, stemness, differentiation, and apoptosis.

In general, miRNA genes are frequently located in cancer-associated genomic regions or fragile sites that are prone to genetic and epigenetic alterations [8]. Altered miRNA expression levels have been reported in most human cancers. In fact, miRNAs can function as oncogenes or as tumor suppressor genes by targeting different steps of the tumorigenesis process, such as initiation, progression, and metastasis [9,10]. Recently, miRNA profiling studies have led to the identification of miRNAs that are aberrantly expressed in breast cancer [11]. A complete identification of mRNA/miRNA expression-based breast cancer subtypes will allow the prediction of prognosis, therapy response, and resistance development.

The goal of this study was to determine the most important miRNAs that are altered in breast tumorigenesis and to find an association between these miRNAs and novel proteins involved in cancer signaling pathways. In this study, we have proposed a breast cancer miRNA signature and have identified novel miRNAs that are related to breast tumorigenesis. According to our results, miR-125b was the most downregulated miRNA in breast tumors. miR-125b represents a paradoxical miRNA because its phenotypic effects differ considerably, depending on cell type. Thus, miR-125b can function as a tumor suppressor miRNA in many tumor types, including ovarian cancer, hepatocellular carcinoma, melanoma, bladder cancer, glioma, colorectal cancer, breast cancer and osteosarcoma [12-19]. However, miR-125b may have a tumor-promoting function in other types of cancer, including prostate cancer [20] and leukemia [21].

Another goal of this study was to functionally characterize the previously reported tumor suppressor role of miR-125b in breast tumorigenesis [18]. Our experiments show that induced expression of miR-125b in cells of mammary origin decreased cell proliferation and anchorage-independent cell growth. Accordingly, we show for the first time that miR-125b performs its antiproliferative function by directly binding to the 3’-UTR mRNAs of several uncharacterized genes, such as cyclin J (CCNJ) and multiple EGF-like-domains 9 (MEGF9). Moreover, miR-125b also downregulates other proteins whose roles in tumorigenesis are not well defined, such as glutamyl aminopeptidase or aminopeptidase A (ENPEP), and casein kinase 2-alpha (CK2-α). Finally, we analyzed the protein expression of ENPEP, CK2-α, CCNJ, and MEGF9 in breast cancer patients. The prognostic role of ENPEP, CK2-α, CCNJ, and MEGF9 proteins and their importance in breast tumorigenesis are examined.

## Materials and Methods

### Patients

Written informed consent was obtained from each patient for the analysis of both normal and tumor tissue. All of the procedures performed in this study were approved by the Ethics Committee of Vall d’Hebron Hospital. All of the patients included in this study had been diagnosed with sporadic breast cancer, and they were followed for a minimum of 2–3 years. No patients in this study had been previously treated with chemo-/radiotherapy prior to surgery, and the primary tumors were isolated in all cases. For each patient, the following pathological parameters were studied: age (<60 versus >60), tumor size, tumor grade, the presence and number of lymph node metastases, and the presence of distant metastases. The molecular classifications of the tumors were luminal A (42%), luminal B (38%), HER2+ (6%), and triple negative (14%).

A comprehensive miRNA profiling study was performed with frozen normal and tumor tissues from 50 patients with breast cancer (series 1). The tumor slides from all patients included in this study were stained with hematoxylin-eosin and were independently reviewed by pathologists to ensure that the tissues, which had been stored at -80°C, had adequate (>80%) tumor densities. Normal tissues from the same patients were stained with hematoxylin-eosin to ensure an adequate content of epithelial cells. Normal tissue could not be obtained from all 50 patients who were selected for our study. Therefore, normal tissue samples from a total of 24 out of 50 patients (pools: A, B and C; each pool comprised 8 patients) were analyzed.

An independent series of 50 additional patients (series 2), who were sorted into pools, was also studied. Two pools of miRNA samples were obtained from the second series of patients. One pool was from breast cancer tissue (50 patients) and the other pool was from the available normal tissue (20 out of 50 patients). Moreover, 25 additional breast cancer patients with similar pathological characteristics were selected for miRNA and protein expression analysis (series 3). In series 3, normal tissue was available for all 25 patients.

The biopsies were retrieved from the Tumor Bank in the Pathology Department of Vall d’Hebron Hospital and diagnosed at the Vall d’Hebron Hospital between 2007 and 2009.

### RNA isolation, reverse transcription, and quantitative real-time polymerase chain reaction

The frozen normal and tumor tissues were collected for RNA extraction. The total RNA from the different breast cancer cells used for this study and the patient biopsies were isolated with the mirVana kit (Ambion, Austin, TX, USA) according to the manufacturer’s instructions. Reverse transcription was performed by using total RNA with stem-loop RT specific primers for miR-125b or endogenous control small nuclear RNU24 (RNU24) and the TaqMan MicroRNA Reverse Transcription kit (Applied Biosystems, Foster City, CA, USA). miRNA expression was measured by quantitative real-time polymerase chain reaction (qRT-PCR) with a specific TaqMan MicroRNA Assay (20X; forward primer, reverse primer, and probe) for miR-125b (ID: 000449) and RNU24 endogenous control (ID: 001001) and assayed on an ABI Prism 7500 System (Applied Biosystems) with cycling conditions of 95°C for 10 min, followed by 40 cycles of 95°C for 15 s and 60°C for 60 s. The qRT-PCR was conducted in triplicate. The Ct (cycle threshold) values of RNU24 were not different between the tumors and the normal breast tissue samples (as detected by qRT-PCR). The Ct values also did not differ among the cell lines studied (data not shown). Relative expression of miR-125b normalized to RNU24 was calculated with the 2^-ΔΔCt^ method.

### miRNA microarray preparation

The human miRNA arrays (Agilent, Santa Clara, CA, USA) containing 13,737 probes for 939 miRNAs were hybridized with the miRNA fraction from the normal and tumor tissues of 50 breast cancer patients (series 1). Briefly, 500 ng of total RNA from each sample was chemically labeled by dephosphorylation with calf intestinal alkaline phosphatase (CIP) and ligation of cyanine3 (pCp) with T4-RNA ligase by using the Agilent miRNA Complete Labeling and Hyb Kit (p/n5190-0456; Agilent). The labeled RNA samples were dried, resuspended in 18 l of nuclease-free water, and cohybridized with the miRNA array in *in situ* hybridization buffer for 20 h at 55°C. The samples were then washed at room temperature for 5 min in gene expression wash buffer 1 and 5 min at 37°C in gene expression wash buffer 2. Images of the samples were generated on a confocal microarray scanner (G2565BA; Agilent) at 5 µm resolution and quantified with the feature extraction software (Agilent). Array data have been submitted to the Gene Expression Omnibus dataset (accession number GSE44124).

A different miRNA array platform (FEBIT, Heidelberg, Germany) was used to validate our results with an independent series of patients (series 2). The protocols for the sample preparation and analysis of this array platform are described elsewhere [22,23].

### Immunohistochemistry

The CK2-α protein was studied by immunohistochemistry (IHC) in the 50 patient samples included in the miRNA array (series 1). Paraffin-embedded biopsies were studied for IHC, and the IHC study was performed in tissue microarrays (TMAs). The TMAs were conducted in quadruplicate by extracting tissue cores from the original paraffin-embedded biopsies as previously described [23]. Paraffin-embedded, formalin-fixed tissue was cut into 5 µm sections, placed on polylysine-coated slides, deparaffinized in xylene, and rehydrated through a graded ethanol series. Endogenous peroxidase activity was quenched in 0.3% hydrogen peroxide, and the tissue was subjected to antigen retrieval treatment by microwave heating in 10 mM citrate buffer (pH 6.0). The sections were incubated at 4°C overnight with the CK2-α antibody (Bethyl Laboratories, Montgomery, TX, USA). The immunostaining was performed with the ChemMate DAKO EnVision Detection Peroxidase/DAB kit (Dako, Glostrup, Denmark), which resulted in a brown-colored precipitate at the antigen site. Subsequently, the sections were counterstained with hematoxylin-eosin and mounted in nonaqueous mounting medium (Zymed Laboratories, San Francisco, CA, USA). CK2-α was validated by using the histoscore system, as previously described for other antibodies [22]. CK2-α expression was considered positive when more than 50% of the tumor cells in the biopsy stained positive (score > 60).

### Cell culture

Human mammary epithelial cells (HMEC) were obtained from Lonza (Lonza, Basel, Switzerland). MCF7, MDA-MB-231, and MDA-MB-435 cells were obtained from the American Type Culture Collection (ATCC, Manassas, VA, USA). The HMEC were grown in mammary epithelium basal media (Lonza) supplemented with MEGM SingleQuots (Lonza). The MCF7 and MDA-MB-435 cells were grown in Dulbecco’s Modified Eagle’s Medium (Lonza), and the MDA-MB-231 cells were grown in RPMI-1640 medium (Lonza). Both media were supplemented with 10% fetal bovine serum, 100 U/ml penicillin, and 100 µg/ml streptomycin. All of the cells were grown at 37°C in a humidified incubator with 5% CO_2_. The cells were passaged regularly at subconfluence.

### Plasmid construction

For the miR-125b overexpression experiments, pre-miR-125b was cloned into retroviral vector miR-Vec, which was kindly donated by Dr. R. Agami (Netherlands Cancer Institute, Amsterdam). Approximately 500 nt of the genomic DNA sequence that encodes for primary miR-125b and its natural flanking sequences was selected for PCR amplification, according to a previously described procedure [24]. Primers are detailed in Table S1.

For the luciferase assay, a reporter vector consisting of the luciferase coding sequence followed by the wild-type or mutant 3’-UTR of putative targets of miR-125b was constructed. The 3’-UTRs of ENPEP (945 nt), CK2-α (1245 nt), CCNJ (916 nt), and MEGF9 (2061 nt), which contain the predicted miR-125b binding sites, were amplified by PCR from the normal tissues and cloned into the previously described pCI vector [25]. Primers are described in Table S1. In all cases, the cloned PCR products were validated by sequencing (data not shown).

### Site-directed mutagenesis

The QuikChange II XL Site-Directed Mutagenesis Kit (Stratagene, La Jolla, CA, USA) was used to introduce point mutations into the 3’-UTRs of human ENPEP, CK2-α, CCNJ, and MEGF9. The following point mutations were introduced: at position 202 of the 3’-UTR-ENPEP, at positions 210 and 450 of the 3’-UTR-CK2-α, at position 714 of the 3’-UTR-CCNJ, and at three different positions—416, 654, and 1721—of the 3’-UTR-MEGF9. A different mutant plasmid was constructed for each binding site located at the 3’-UTR mRNA predicted for miR-125b. Primers are described in Table S1.

The PCR reaction was prepared by adding 5 µl of 10X reaction buffer, 20 ng of dsDNA template, 125 ng each of the sense and antisense primers, 1 µl of deoxyribonucleotide triphosphate mix, 3 µl of QuickSolution, 1 µl of *Pfu* Ultra HF DNA polymerase (2.5 U/µl), and double-distilled water to a final volume of 50 µl. The PCR was performed with 18 cycles (95°C for 50 s; 60°C for 50 s; and 68°C for 1 min/kb of the plasmid length), including an initial incubation at 95°C for 1 min and a final extension period at 68°C for 7 min. After the PCR reaction, 1 µl of the *Dpn*I restriction enzyme (10 U/µl) was added directly to each amplification reaction and incubated at 37°C for 1 h to digest the parental supercoiled dsDNA. The *Dpn*I-treated dsDNA was transformed into XL10-Gold Ultracompetent cells. The colonies selected were expanded in culture for DNA extraction and the presence of the mutation was confirmed by subsequent sequencing (data not shown).

### Bioinformatics searches

Potential miR-125b targets were predicted and analyzed by using publicly available algorithm-based databases, including PicTar (http://pictar.mdc-berlin.de/), TargetScan (http://www.targetscan.org/), miRanda (http://www.microrna.org/), and DIANA-microT (http://diana.cslab.ece.ntua.gr/). To select the putative miR-125b mRNA targets, two different criteria were used. The first criterion consisted of selecting mRNAs that are predicted to be targets of miR-125b by more than one miRNA database. Of these ENPEP, CK2-α, and MEGF9 were selected. The second criterion consisted of selecting mRNAs with a high score (*p* value) according to the TargetScan database. Based on the latter criterion, CCNJ was selected. For both criteria, those mRNAs that may be involved in cell proliferation were considered for protein studies.

### Transfection

For the stable expression of miRVec-125b or miRVec-GFP (control cells), 30 µg of each retroviral vector was transfected into Phoenix cells in 10-cm culture plates with FuGENE (Roche, Basel, Switzerland). The viral supernatant was harvested 48 h after transfection to infect HMEC, MCF7, and MDA-MB-231 cells. Each stably transduced cell line was selected with blasticidin (3 µg/ml for HMEC, 10 µg/ml for MCF7, and 10 µg/ml for MDA-MB-231) for 12 days.

For anti-miR-mediated silencing of miR-125b, MDA-MB-435 and MCF7 cells were seeded at 2.5 × 10^5^ and 2.0 × 10^5^ cells per well, respectively, in 6-well plates and transiently transfected with anti-125b (ID: AM10148; Ambion) or a Cy3 dye-labeled negative control (ID: AM17010; Ambion) to a final concentration of 80 nM with the HiPerFect reagent (Qiagen, Hilden, Germany) according to the manufacturer’s instructions. At 72 h after transfection, the cells were counted and imaged, and the cellular lysates were collected for analysis of the protein expression of the selected putative miR-125b targets. The transfection efficiency was determined by fluorescence microscopy with the Cy3 dye-labeled negative control.

The knockdown experiments for ENPEP, CK2-α, CCNJ, and MEGF9 were performed in MCF7 cells. MCF7 cells were seeded at 2 × 10^5^ cells per well in a 6-well plate and were transiently transfected with pools of siRNAs (siGENOME SMARTpool) against ENPEP (M-005865-01-0005), CK2-α (M-003475-03), CCNJ (M-020397-01-0005), and MEGF9 (M-026241-01-0005) or a pool of negative control siRNAs (siGENOME Non-Targeting siRNA Pool #1; 0-001206-13-05) (Dharmacon, Lafayette, CO, USA) at a final concentration of 30 nM with the HiPerFect transfection reagent (Qiagen) according to the manufacturer’s protocol. Each siRNA pool contained a mixture of three different siRNAs for each gene. To control for transfection efficiency, in addition to the Cy3-labeled control siRNA, polo-like kinase 1 (PLK1) siRNA (ON-TARGET plus SMART pool; Dharmacon L-003290-00-0005) was also included (data not shown). The transfected cells were incubated at 37°C for 72 h, counted, and collected for protein extraction.

To study the long-term effects of gene silencing, MCF7 cells were seeded at 2 × 10^5^ cells per well in a 6-well plate and stably transfected the following day with 3 µg of puromycin-resistant SureSilencing™ shRNA plasmids. For each gene, four different shRNAs were tested independently (No. 1, No. 2, No. 3, and No. 4). The shRNAs for ENPEP (ID: KH02578P; Qiagen), CK2-α (ID: KH01514P; Qiagen), CCNJ (ID: KH15834P; Qiagen), MEGF9 (ID: KH14779P; Qiagen), and the negative control shRNA plasmid NEG1-P (Sc) (ID: KA-001P; Qiagen) were transfected with jetPEI transfection reagent (Genycell, Granada, Spain) according to the manufacturer’s instructions. To generate stably transfected cells, MCF7 cells were selected with puromycin (1 µg/ml) (Sigma-Aldrich, St. Louis, MO, USA) for 3 days. The growth curve analyses and protein extractions were performed as previously described [22]. Parental MCF7 cells (uninfected/untransfected) were included in all experiments.

### Growth curves

HMEC, MCF7, and MDA-MB-231 cells were seeded at 1 × 10^6^ cells per 10-cm plate. Parental cells for each cell line (uninfected), the control cells (miRVec-GFP-infected), and cells that expressed miRVec-125b were grown simultaneously. Every 3 days, the cells from each cell line were counted and reseeded at a density of 1 × 10^6^ cells per 10-cm plate, as indicated by the 3T3 protocol. In addition, 5 × 10^4^ cells per well were reseeded every 3 days in 24-well plates in triplicate and fixed. Cell staining was performed with crystal violet.

### Soft agar colony formation assay

For the soft agar colony formation assay, MCF7 and MDA-MB-231 cells that expressed the control miRVec-GFP or miRVec-125b were seeded in triplicate at a density of 5 × 10^4^ cells/well in 6-well plates that contained medium with 1.4% D-1 Low EEQ agarose (Pronadisa, Madrid, Spain). The culture medium was changed every 3 days, and the colonies were counted and imaged after 20 days in culture.

### Cell cycle analysis

For the cell cycle analysis, a fluorescence-activated cell sorting Calibur flow cytometer (FACS Calibur Becton Dickinson, E0772; BD Biosciences, San Jose, CA, USA) was used to analyze HMEC, MCF7, and MBA-MB-231 cells that stably expressed control miRVec-GFP or miRVec-125b. The siRNA- and shRNA-transfected cells (si/shENPEP, si/shCK2-α, si/shCCNJ, and si/shMEGF9) were also analyzed by FACS. One million cells from each sample were fixed in 70% ethanol for 15 min at -20°C, treated with 100 µg/ml RNase A (Sigma-Aldrich), and stained with 50 µg/ml of propidium iodide (Sigma-Aldrich). For each sample, 2 × 10^4^ cells were analyzed, and the percentage of cells in each phase of the cell cycle was calculated based on the DNA content determined with FACS Express Software.

### Annexin V-APC apoptosis analysis

HMEC, MCF7, and MDA-MB-231 cells were infected with miRVec-125b or miRVec-GFP and were selected with blasticidin for stable expression. The analysis of apoptotic cells was performed with the Annexin V-APC Detection Kit (eBioscience, San Diego, CA, USA) according to the manufacturer’s instructions. The samples were then analyzed by FACS. For each sample, 2 × 10^4^ cells were collected and the results were analyzed with the FACS Express Software. The siRNA- and shRNA-transfected cells (si/shENPEP, si/shCK2-α, si/shCCNJ, and si/shMEGF9) were also analyzed for the presence or absence of apoptosis. The results were confirmed in at least three independent experiments.

### Analysis of senescence

Senescence-associated β-galactosidase (SA--gal) activity was analyzed with a senescence β-galactosidase commercial assay according to the manufacturer’s instructions (Cell Signaling, Danvers, MA, USA), as previously described [26]. Senescence controls were included as indicated.

### Luciferase reporter assay

The luciferase experiments were performed in HEK293T cells. HEK293T cells were seeded at 1.4 × 10^5^ cells per well in a 24-well plate and were transfected the following day with Lipofectamine 2000 (Invitrogen, Carlsbad, CA, USA) with the following molecules: the synthetic miRNA precursors to miR-125b (miR-125b mimics) (ID: PM10148; Ambion), anti-125b (ID: PM10149; Ambion), or scrambled negative control 1 (ID: AM17110; Ambion); and the wild-type luciferase constructs cloned in the pCI plasmid: pCI versus pCI-3’-UTR-ENPEP, pCI-3’-UTR-CK2-α, pCI-3’-UTR-CCNJ, or pCI-3’-UTR-MEGF9; Renilla (PGL3-Pm) for normalization of transfection efficiency; and pBluescript for DNA content normalization. The transfection efficiency was approximately 95%, and luciferase activity was measured 48 h after transfection with the dual-luciferase reporter assay as described by the manufacturer (Promega, Fitchburg, WI, USA).

In each case, the different miR-mimics/anti-miR concentrations were measured by titrating the miR-mimics/anti-miR with each pCI-3’-UTR mRNA construct to establish a dose-response relationship between 10–80 nM (data not shown). Moreover, transfection of mutant luciferase constructs—pCI-M202-3’-UTR-ENPEP, pCI-M210-3’-UTR-CK2-α, pCI-M450-3’-UTR-CK2-α, pCI-M714-3’-UTR-CCNJ, pCI-M416-3’-UTR-MEGF9, pCI-M654-3’-UTR-MEGF9, pCI-M1721-3’-UTR-MEGF9—or their respective wild-type pCI-3’-UTR mRNA was performed with miR-125b mimics or scrambled negative control in HEK293T cells. All transfection experiments were conducted in triplicate and repeated at least 3 times independently.

### Immunoblotting

Total cell lysates were prepared from a confluent 10-cm dish. The cells were washed in PBS and lysed in 1 ml of lysis buffer (50 mM Tris-HCl, pH 7.5, 1% NP-40, 10% glycerol, 150 mM NaCl, plus 2 mM complete protease inhibitor cocktail) (Roche Diagnostics, Barcelona, Spain). From each sample, 50 µg of protein, which was previously quantified with the Bio-Rad protein assay (Bio-Rad, Hercules, CA, USA), was then analyzed by gel electrophoresis on a 6–12% SDS-polyacrylamide gel and transferred onto a nitrocellulose membrane. The following antibodies were used for western blot analysis: ENPEP (Novus Biologicals, Littleton, CO, USA), MEGF9 (Abcam, Cambridge, UK), CK2-α (Bethyl Laboratories), CCNJ (Santa Cruz Biotechnology, Santa Cruz, CA, USA), CCND1 (Santa Cruz Biotechnology), CCNB1 (Santa Cruz Biotechnology), and -actin (Sigma-Aldrich). In all cases, the membranes were incubated overnight at 4°C with the primary antibodies in PBS with 5% nonfat dry milk. The membranes were then extensively washed with PBS and incubated with horseradish peroxidase-conjugated anti-mouse or anti-rabbit secondary antibody (Sigma-Aldrich). After additional washes with PBS, the antigen-antibody complexes were visualized with an enhanced chemiluminescence kit (Millipore, Billerica, MA, USA).

### Statistical analysis

Data analysis was performed with SPSS statistical software (version 11.0; SPSS, Chicago, IL, USA). The analysis of the miRNA array was performed as previously described [27] (*p* < 0.05 was considered significant). To establish the miRNA signature, miRNAs that were differentially expressed between the two classes of tissues were compared with a *t*-test (*p* < 0.01 was considered significant). To compare the expression of miR-125b by qRT-PCR in normal and tumor tissues, a Mann-Whitney test or Wilcoxon test was used as appropriate. Luciferase experiments were analyzed by a Student’s *t*-test. Lastly, a Pearson’s correlation test was used to compare the pathological variables with the levels of ENPEP, CK2-α, CCNJ, and MEGF9 protein expression and miR-125b expression (*p* < 0.05 was considered significant).

## Results

### miRNA expression profile in breast tumors

**Figure 1:**
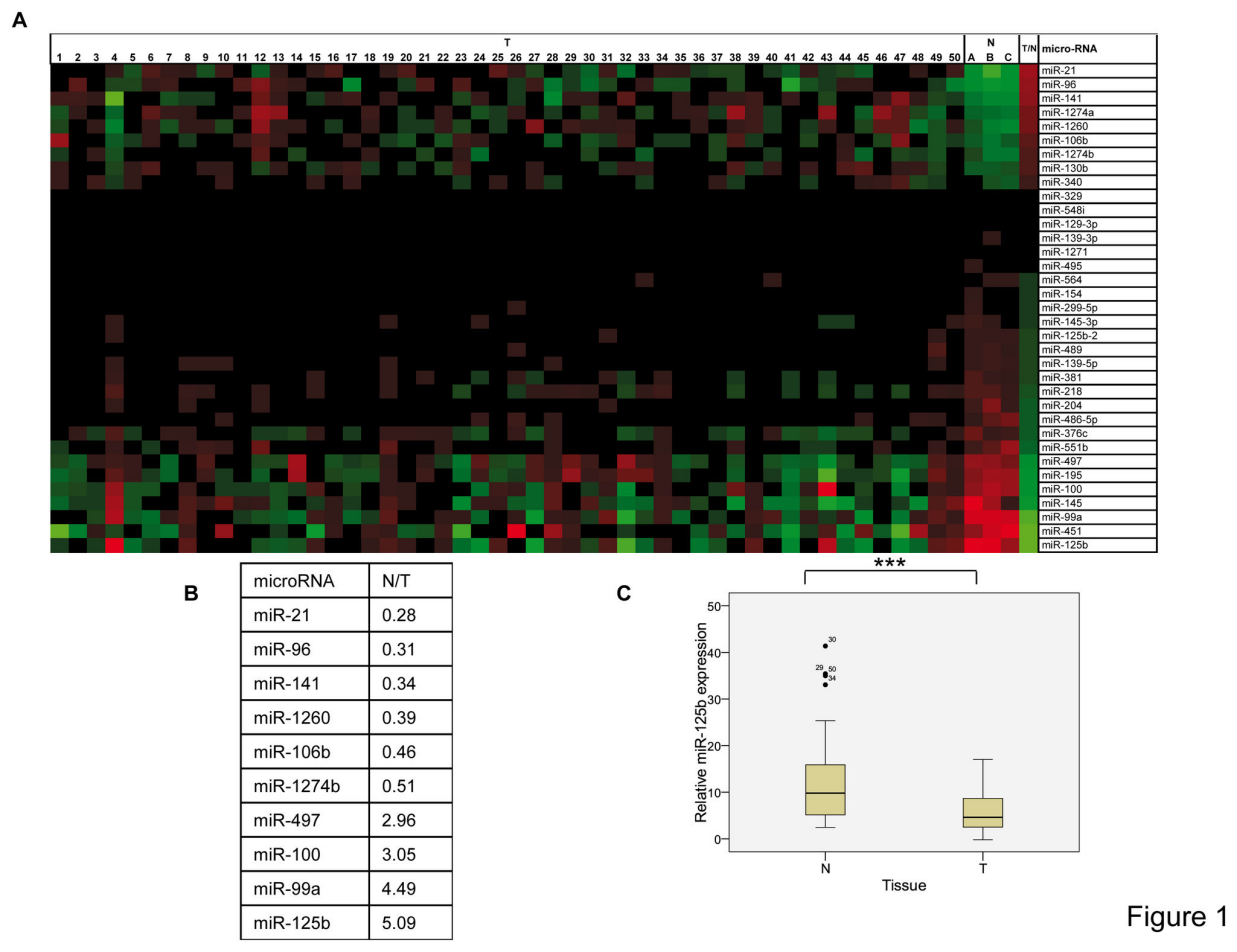
The miRNA array in breast cancer patients. (**A**) A total of 35 significantly dysregulated miRNAs were differentially expressed in tumor (T) and normal (N) breast tissues (pool A and pool B) (p < 0.05) (see Materials and Methods). (**B**) The miRNA signature of differentially expressed miRNAs in breast cancer patients. To establish the miRNA signature, miRNAs that were differentially expressed between the two classes of tissues were compared with a *t*-test (p < 0.01). N/T: The numbers indicate the mean fold up- or downregulation in the normal tissue with respect to the tumor tissue. The most significantly deregulated miRNA is miR-125b, which shows more than a 5-fold downregulation in tumor tissue. (**C**) qRT-PCR of miR-125b in the patients analyzed in Figure 1A. The standard deviation is indicated. ***p < 0.001.

A comprehensive panel of 939 miRNAs (Agilent) was analyzed in 50 breast cancer tumor samples, which were compared with three pools of normal breast tissue from the same patients (series 1; see Materials and Methods). A total of 35 miRNAs were dysregulated in the tumor sample compared with normal breast tissue (Figure 1A, Table S2). These miRNAs comprise two different classes, namely oncogenic and tumor suppressor miRNAs. The 9 putative oncogenic miRNAs were miR-21, miR-96, miR-141, miR-1274a, miR-1260, miR-106b, miR-1274b, miR-130b, and miR-340. The 26 putative tumor suppressor miRNAs were miR-125b, miR-451, miR-99a, miR-145, miR-100, miR-195, miR-497, miR-551b, miR-376c, miR-486-5p, miR-204, miR-218, miR-381, miR-139-5p, miR-489, miR-125b-2, miR-145-3p, miR-299-5p, miR-154, miR-564, miR-495, miR-1271, miR-139-3p, miR-129-3p, miR-548i, and miR-329. To establish the minimum number of miRNAs that distinguish the tumors from normal breast tissues, an “miRNA signature” was established by assessing 10 miRNAs (Figure 1B).

To confirm our proposed “miRNA signature”, we used a different series of patients (series 2) and a distinct array platform (FEBIT). All miRNAs were also confirmed in the second series of patient samples, with the exception of miR-21 (Table S3). Moreover, in this second series of patient samples, miR-125b was observed to be significantly downregulated (Table S3).

To independently validate miR-125b downregulation, the levels of miR-125b in breast cancer patient samples from series 1 were also analyzed by qRT-PCR (Figure 1C). The expression of miR-125b was compared with the pathological characteristics of the breast cancer patients, including age, tumor size, grade of differentiation, presence of lymph node metastases, number of affected nodules, presence of distant metastases, and the molecular classification of the tumors as described (luminal A, luminal B, HER2+, and triple negative) [28]. Because only 6% of tumors were HER2+ and 14% of tumors were triple negative in our series (series 1), we consider the results regarding molecular classification to be preliminary (data not shown). For statistical conclusions regarding the molecular classification of the tumors, a larger series of patients would be required to increase the number of these infrequent tumor subtypes (i.e., HER2+ and triple negative). Overall, miR-125b did not correlate with any pathological characteristics and was confirmed to be an miRNA consistently downregulated in breast cancer patients. Therefore, we focused on further characterization of miR-125b in breast cancer tumor suppression.

### miR-125b decreases cell proliferation and anchorage-independent cell growth in mammary cells

**Figure 2:**
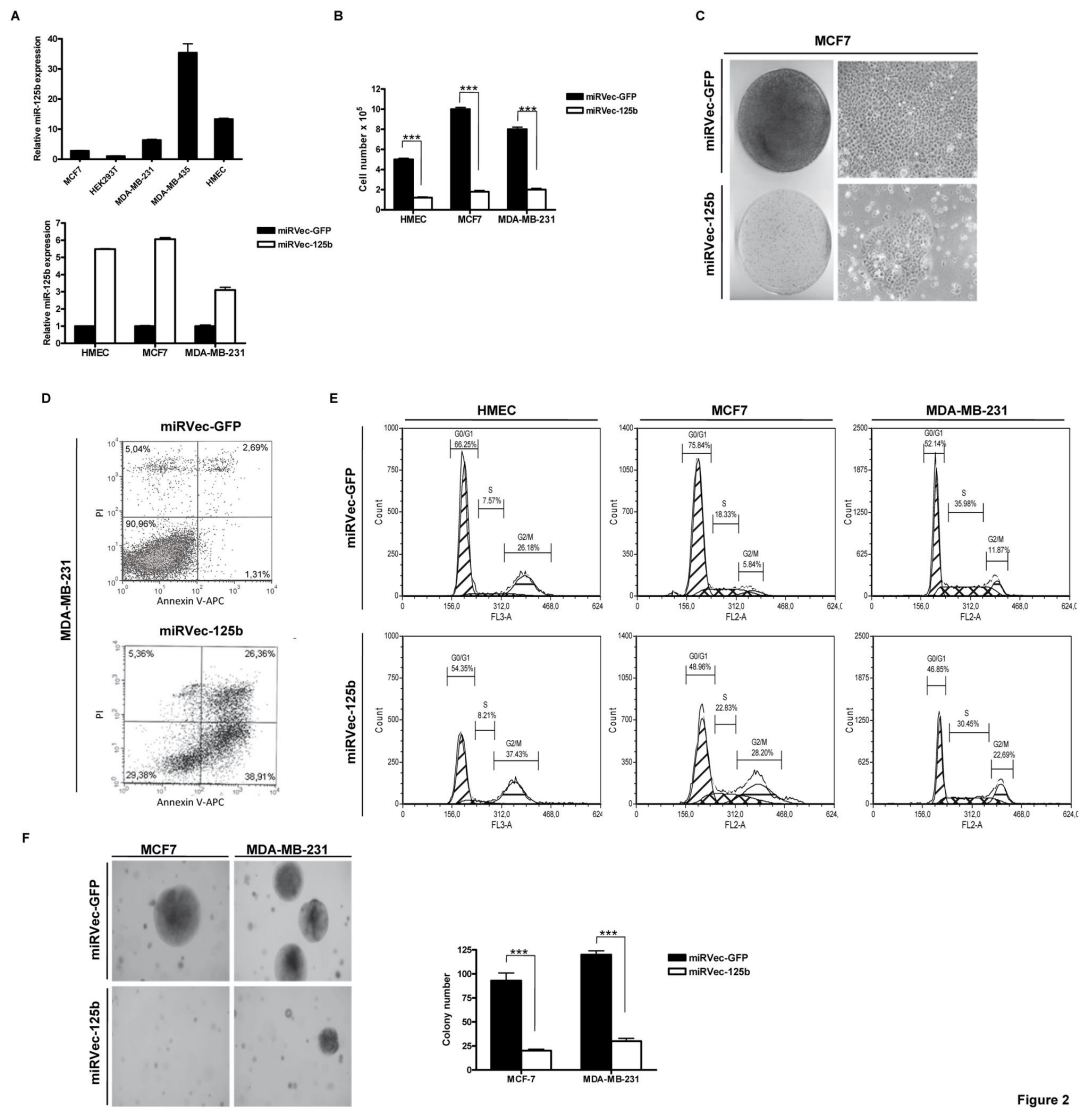
miR-125b expression decreases cell proliferation and anchorage-independent cell growth in mammary cells. (**A**) Upper panel: miRNA expression levels of miR-125b assessed by qRT-PCR in different cell lines. The graph shows data normalized to RNU24 levels. The relative expression of miR-125b normalized to RNU24 was calculated with the 2^-ΔΔCt^ method. HEK293T cells were assigned the value of 1 as they express the lowest relative RNA level of miR-125b in comparison with the other cells lines shown. Lower panel: miRNA expression levels of miR-125b assessed by qRT-PCR in different cell lines. miRVec-125b-overexpressing cells compared to miRVec-GFP control cells in MCF7, MDA-MB-231, and HMEC cells. These cell lines were pooled heterogeneous clones of the indicated genes after stable expression and selection with blasticidin for 12 days. (**B**) Cell numbers upon miRVec-125b overexpression compared with those of miRVec-GFP control cells in the indicated cell lines after stable expression and selection of the indicated genes with blasticidin for 12 days. (**C**) Phenotypic morphology of miRVec-GFP- and miRVec-125b-transduced MCF7 cells (right panel) and crystal violet staining of miRVec-GFP- and miRVec-125b-transduced MCF7 cells (left panel). Cells were selected as indicated in B. (**D**) Apoptosis rates analyzed by FACS of miRVec-GFP- and miRVec-125b-transduced MDA-MB-231 cells. Note the increases in early (1.3 ◊ 38.9%) and late (2.6 ◊ 26.3%) apoptosis in miRVec-GFP- and miR-125b-overexpressing cells. (**E**) The cell cycle profiles of miRVec-GFP- and miRVec-125b-transduced HMEC, MCF7, and MDA-MB-231 cells. miRVec-GFP-HMEC (G0/G1, 66.2%; S, 7.6%; G2/M, 26.2%) versus miRVec-125b-HMEC (G0/G1, 54.3%; S, 8.2%; G2/M, 37.4%); miRVec-GFP-MCF7 (G0/G1, 75.8%; S, 18.3%; G2/M, 5.8%) versus miRVec-125b-MCF7 (G0/G1, 48.9%; S, 22.8%; G2/M, 28.2%), and miRVec-GFP-MDA-MB-231 (G0/G1, 52.1%; S, 36%; G2/M, 11.9%) versus miRVec-125b-MDA-MB-231 (G0/G1, 46.9%; S, 30.5%; G2/M, 22.7%) (**F**) Representative images of colonies of the soft agar colony assay in miRVec-GFP- and miRVec-125b-transduced MCF7 cells. ***p < 0.001.

To thoroughly investigate the biological effects of the most significantly dysregulated miRNA in breast tumors, miR-125b was stably overexpressed in MCF7, MDA-MB-231, and HMEC cell lines, which have varying degrees of malignancy. This experiment was performed to determine whether the biological effect of miR-125b differs between normal and tumorigenic cells. First of all, we analyzed by qRT-PCR the endogenous expression level of miR-125b in a panel of cancer cell lines and compared these levels with those of normal HMEC cells (Figure 2A, upper panel). To validate the ectopic expression of miR-125b in the breast cancer cells MCF7, MDA-MB-231, and normal HMEC cells, qRT-PCR was employed to measure the expression levels of miR-125 by comparing miRVec-125b- and miRVec-GFP-infected cells (Figure 2A, lower panel). Parental MCF7 cells (non-infected) were tested in parallel with the infected cells and were found to behave like miRVec-GFP-infected cells (data not shown).

Second, to confirm miR-125b expression, we also analyzed proteins whose corresponding 3’-UTR mRNAs had been previously described to directly interact with miR-125b, such as p53, p-AKT, p-MAPK, and members of the E2F family [15,29,30] (Figure S1A and data not shown). Our results confirm the downregulation of p53 (the best known miR-125b target [31]) in the MCF7 and HMEC cells infected with miRVec-125b in comparison with miRVec-GFP-infected cells (which have wild-type p53). However, no differences in p53 levels were observed in MDA-MB-231 cells (mutant p53) (data not shown). As expected, other proteins related to the p53 pathway, such as p21^CIP1^ and HDM2, were also affected in MCF7 and HMEC cells (Figure S1A and data not shown). No differences in p-AKT, p-MAPK, E2F1, and E2F2 were observed in any cell line included in our study (Figure S1A and data not shown).

After establishing the miR-125b expression levels in MCF7, MDA-MB-231, and HMEC cells, we examined the cell proliferation capability related to miR-125b. Our results showed that overexpression of miR-125b significantly decreased cell proliferation (Figure 2B and 2C, Figure S1B, and data not shown). To identify the potential mechanism responsible for the observed effects of miR-125b on cell proliferation, we performed senescence and cell cycle analysis and evaluated apoptosis rates in the three cell lines transduced with miRVec-125b or miRVec-GFP. For all three cell lines, the decrease in cell proliferation was not due to a senescence phenotype because we did not observe senescence-associated -galactosidase activity (Figure S1C and data not shown). Ectopic expression of miRVec-125b induced G2/M cell cycle arrest in selected cell lines (Figure 2E). In addition to the increased number of cells accumulating in the G2/M phase, miRVec-125b overexpression induced apoptosis in MDA-MB-231 cells (Figure 2D and Figure S2A). In contrast, there were no differences in the apoptosis rates between cells overexpressing miRVec-125b and miRVec-GFP in MCF7 and HMEC cell lines (Figures S2A and S2B).

On the other hand, to determine the putative tumor suppressor role of miR-125b, we tested its ability to reverse transformation by performing soft agar colony transformation assays. Our results showed that overexpression of miR-125b decreased colony numbers by the order of 80% and 75%, respectively, in MCF7 and MDA-MB-231 cells expressing miRVec-125b compared with miRVec-GFP (Figure 2F). Therefore, miR-125b dramatically decreased the anchorage-independent cell growth ability of MCF7 and MDA-MB-231 cells.

### miR-125b directly targets ENPEP, CK2-α, CCNJ, and MEGF9

**Figure 3:**
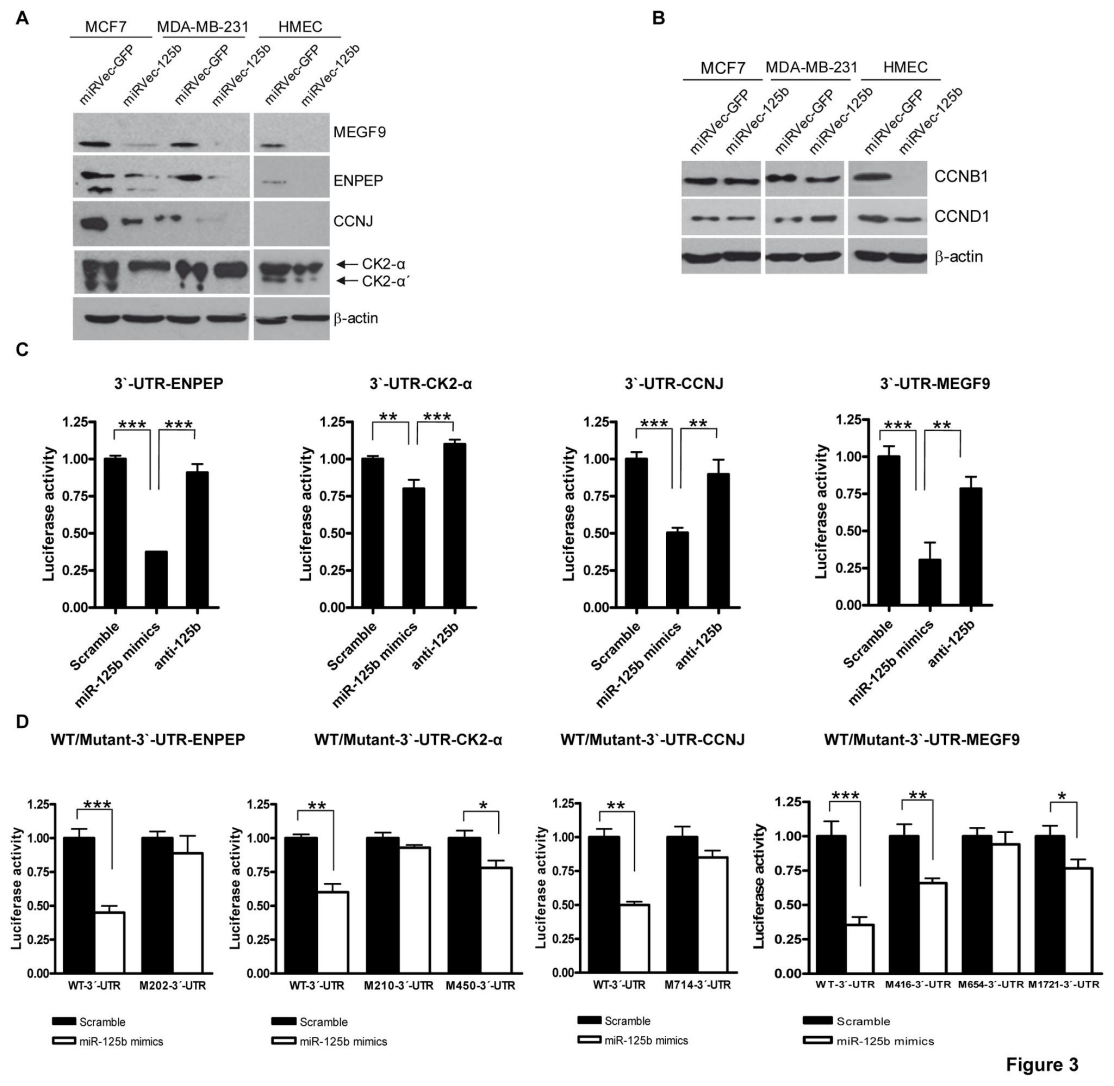
miR-125b directly targets ENPEP, CK2-α, CCNJ, and MEGF9. (**A**) Western blot analysis of MEGF9, ENPEP, CCNJ, and CK2-α (subunits α and α’) in miRVec-GFP- and miRVec-125b-transduced MCF7, MDA-MB-231, and HMEC cells. (**B**) Western blot analysis of cell cycle proteins CCNB1 and CCND1 in miRVec-GFP- and miRVec-125b-transduced MCF7, MDA-MB-231, and HMEC cells. (**C**) The relative luciferase activity of HEK293T cells transfected with miR-125b mimics, anti-125b, or scrambled control in the presence of the wild-type luciferase pCI-3’-UTR constructs of ENPEP, CK2-α, CCNJ, and MEGF9 mRNAs. HEK293T cells were transfected with Lipofectamine 2000 and the final concentration of the scramble, miR-125b mimics, and anti-125b was 30 nM. The luciferase plasmids and the pCI or pCI-3’-UTR for each indicated gene were used at a final concentration of 100 ng; the Renilla plasmid was used at 10 ng. (**D**) The relative luciferase activity of HEK293T cells transfected with miR-125b mimics or scrambled control in the presence of the luciferase constructs containing the wild-type or mutant form of 3’-UTR-ENPEP, 3’-UTR-CK2-α, 3’-UTR-CCNJ, and 3’-UTR-MEGF9 mRNAs (*p < 0.05, **p < 0.01, ***p < 0.001).

By using major prediction software (PicTar, TargetScan, miRanda, and DIANA-microT), the potential binding sites of miR-125b in the 3’-UTR mRNAs of ENPEP, CK2-α, CCNJ, and MEGF9 were predicted. Next, we sought to analyze whether miR-125b is able to regulate the expression of these proteins. Western blotting analysis revealed that the levels of the predicted target proteins ENPEP, CK2-α, CCNJ, and MEGF9 were decreased in miRVec-125b-overexpressing MCF7, MDA-MB-231, and HMEC cells, with the exception that CCNJ is not expressed in HMEC cells (Figure 3A). Of note, in the HMEC cells, the cell cycle proteins CCND1 and CCNB1 were downregulated compared to MCF7 and MDA-MB-231 cells (Figure 3B).

To determine whether the ENPEP, CK2-α, CCNJ, and MEGF9 proteins were downregulated because their 3’-UTR mRNAs were direct targets of miR-125b, a luciferase assay was performed in HEK293T cells, which express low levels of endogenous miR-125b (Figure 2A, upper panel). Therefore, this cell model may clearly reflect the biological effects of the expression of miR-125b mimics. For this purpose, we constructed a reporter vector consisting of the luciferase coding sequence followed by the wild-type or mutant 3’-UTR of the putative targets of miR-125b and used two different cotransfection approaches (see Materials and Methods; Figure S4).

First, we studied the effect of miR-125b mimics and anti-125b on the wild-type pCI-3’-UTR constructs of ENPEP, CK2-α, CCNJ, and MEGF9. In all cases, cotransfection experiments showed that luciferase expression in those constructs containing the wild-type form of 3’-UTR-ENPEP, 3’-UTR-CK2-α, 3’-UTR-CCNJ, and 3’-UTR-MEGF9 was significantly decreased upon transfection of miR-125b mimics, but not the negative control (Figure 3C). More importantly, similar effects on any of the wild-type pCI-3’-UTR mRNA constructs were not observed in the presence of anti-125b (Figure 3C). To confirm the inhibitory function of anti-125b on endogenous miR-125b expression in HEK293T cells, qRT-PCR was performed. Our results showed that anti-125b inhibited miR-125b expression efficiently in a dose-dependent manner (Figure S3B).

Second, we cotransfected miR-125b mimics or scrambled negative control with each luciferase construct containing the wild-type or mutant 3’-UTR mRNAs. Our results showed that miR-125b mimics significantly decreased the luciferase activity of all constructs containing the wild-type 3’-UTR mRNAs compared to control cells, but there was no decrease in luciferase activity in cells expressing the luciferase construct-containing mutant 3’-UTR mRNAs of ENPEP and CCNJ (Figure 3D). In the case of the mutant 3’-UTR mRNAs of MEGF9 and CK2-α, luciferase activity did not decrease significantly in cells expressing the specific mutant constructs pCI-M654-3’-UTR-MEGF9 and pCI-M210-3’-UTR-CK2-α, respectively, upon cotransfection with miR-125b mimics (Figure 3D). In each case, the experiments were performed in triplicate. The results of one representative experiment out of 3–4 independent experiments are shown. Overall, our results revealed that miR-125b does indeed repress the expression of ENPEP, CK2-α, CCNJ, and MEGF9 by direct interaction of miR-125b with their respective 3’-UTR mRNAs.

### miR-125b inhibition promotes cell proliferation and upregulation of miR-125b targets

To confirm that miR-125b decreases cell proliferation and causes downregulation of novel targets of miR-125b, namely ENPEP, CK2-α, CCNJ, and MEGF9, functional inhibition of endogenous miR-125b with anti-125b oligonucleotides was performed. For this purpose, we transiently transfected MCF7 and MDA-MB-435 cells with anti-125b or a scrambled negative control. MDA-MB-435 cells were selected because miR-125b levels have been reported to be approximately 20 times higher than in MCF7 and HEK293T cells [32], an observation corroborated by our study (Figure 2A, upper panel).

**Figure 4:**
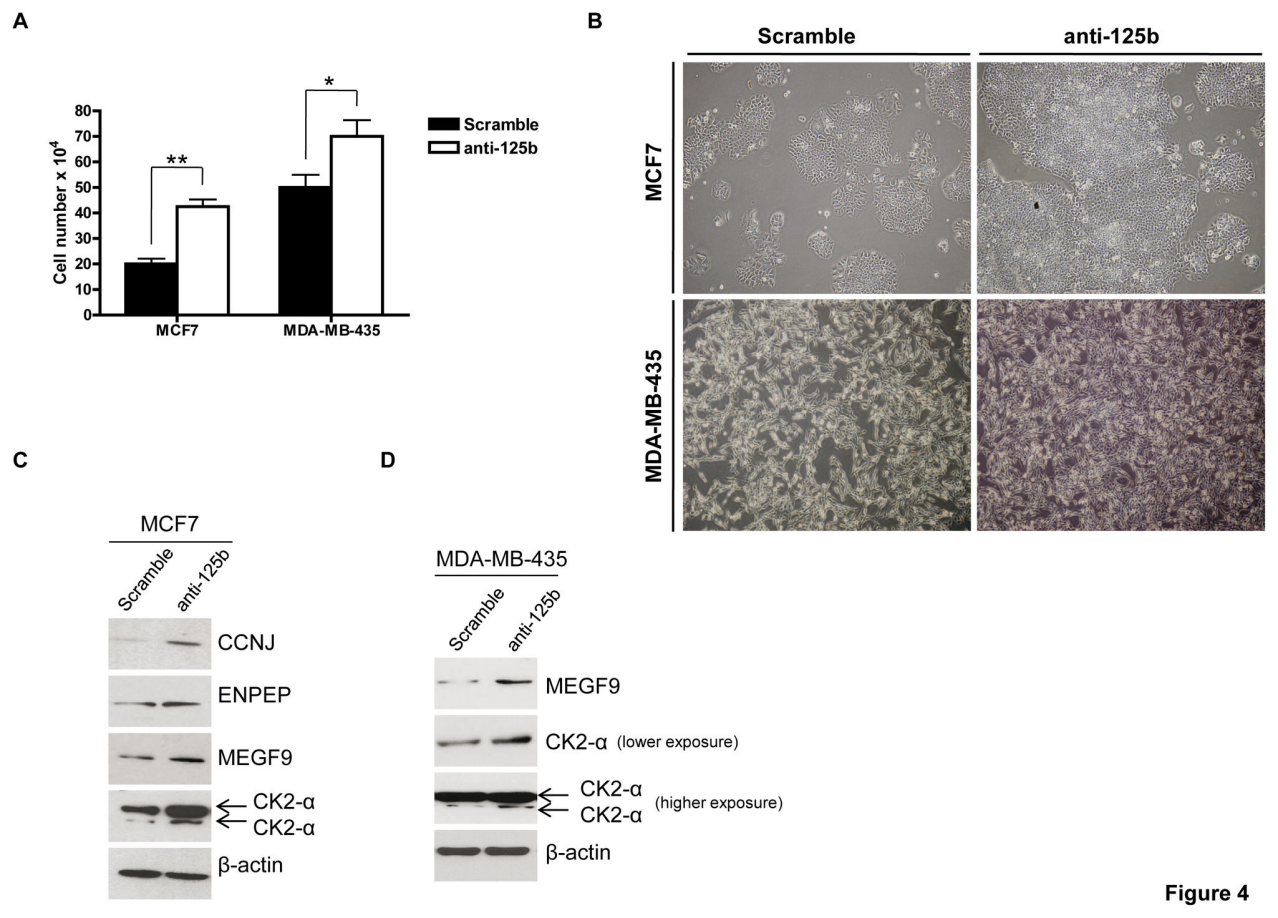
miR-125b inhibition promotes cell proliferation and upregulation of ENPEP, CK2-α, CCNJ, and MEGF9. (**A**) MCF7 and MDA-MB-435 cell numbers after transfection with anti-125b or scrambled control. Cells were transfected with the indicated oligonucleotides at a final concentration of 80 nM each by using HiPerFect reagent. After 72 h of transfection, the cells were counted, photographed, and collected for protein extraction. *p < 0.05, **p < 0.01. (**B**) The phenotypic effects on cell proliferation of anti-125b and scrambled control in MCF7 and MDA-MB-435 cells. The transfection conditions were the same as those described in A. (**C**) Western blot analysis of ENPEP, CK2-α, CCNJ, and MEGF9 in MCF7 cells transfected with anti-125b or scrambled control. The transfection conditions were the same as those described in A. (**D**) Western blot analysis of MEGF9 and CK2-α in MDA-MB-435 cells transfected with anti-125b or scrambled control. The transfection conditions were the same as those described in A.

First, we verified the functionality of the anti-125b molecules in MCF7 and MDA-MB-435 cells. We observed that the inhibitory effect of anti-125b on endogenous miR-125b expression in MCF7 and MDA-MB-435 cell lines was similar to that of HEK293T cells (Figure S3 and data not shown). Moreover, to confirm the effects of anti-125b at the protein level, we checked the expression of a previous validated miR-125b target, p53, in MCF7 cells. p53 protein was upregulated in the presence of anti-125b expression (data not shown). As expected, functional inhibition of endogenous miR-125b significantly increased the proliferation of MCF7 cells compared to the negative control (Figure 4A and 4B). This increase in proliferation was accompanied by an upregulation in the protein expression of the identified novel miR-125b targets, ENPEP, CK2-α, CCNJ, and MEGF9 (Figure 4C). Therefore, anti-125b transfection reversed the effects of miR-125b on cell proliferation and protein expression. In MDA-MB-435 cells, increases in CK2-α (both subunits α and α’) and MEGF9 protein levels were observed, as expected for direct mRNA targets of miR-125b (Figure 4D). Moreover, we observed that MDA-MB-435 cells did not express CCNJ and ENPEP, a fact that may be associated with their high miR-125b levels (data not shown).

### ENPEP, CK2-α, CCNJ, and MEGF9 knockdown recapitulate the biological effects of miR-125b expression

After identifying the 3’-UTRs of ENPEP, CK2-α, CCNJ, and MEGF9 mRNAs as miR-125b targets, we then examined whether repression of these proteins by siRNAs mimicked the effects of miR-125b expression. To understand the contribution of each protein to the proliferation arrest induced by miR-125b expression, the siRNAs targeting ENPEP, CK2-α, CCNJ, and MEGF9, or a scrambled siRNA control, were transiently expressed in MCF7 cells. These siRNAs were transfected into MCF7 cells because the endogenous miR-125b levels were lower in MCF7 cells than other cells lines (Figure 2A, upper panel). Therefore, the levels of some miR-125b targets (such as CCNJ) were higher in MCF7s than in MDA-MB-231 or HMEC cells (which lack CCNJ expression) (Figure 3A).

**Figure 5:**
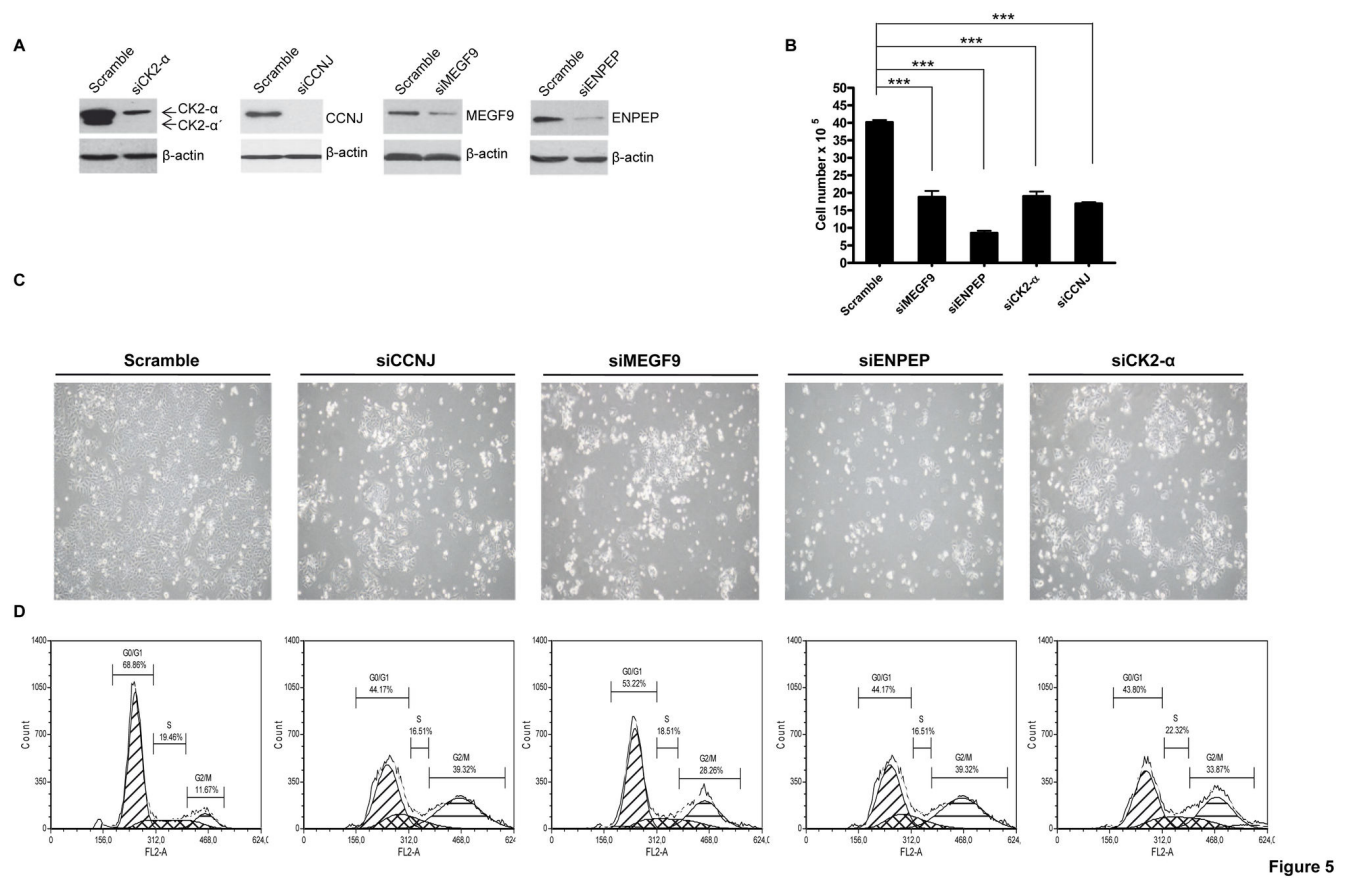
siRNA-mediated ENPEP, CK2-α, CCNJ, and MEGF9 knockdown recapitulates the biological effects of miR-125b expression. (**A**) Western blot analysis of ENPEP, CK2-α, CCNJ, and MEGF9 upon transfection of the indicated siRNAs and scrambled control in MCF7 cells. Cells were transfected with the indicated pools of siRNAs at final concentrations of 30 nM by using HiPerFect transfection reagent. After 72 h of transfection, the cells were counted, photographed, and either collected for protein extraction or trypsinized for FACS analysis. (**B**) Cell numbers after 3 days of transfection with the indicated siRNAs. (**C**) Images of phenotypic effects on cell proliferation after 3 days of transfection with the indicated siRNAs in MCF7 cells. (**D**) The cell cycle profiles of MCF7 cells after expression with the indicated siRNAs or scrambled control. Scramble (G0/G1, 68.9%; S, 19.6%; G2/M, 11.7%), siCCNJ (G0/G1, 44.2%; S, 16.5%; G2/M, 39.2%), siMEGF9 (G0/G1, 53.2%; S, 18.5%; G2/M, 28.2%), siENPEP (G0/G1, 44.1%; S, 16.5%; G2/M, 39.3%), and siCK2-α (G0/G1, 43.8%; S, 22.3%; G2/M, 33.9%). Cells were transfected according to the conditions described in A. ***p < 0.001.

The effectiveness and specificity of each siRNA in reducing the protein levels upon 72 h of transfection were initially confirmed by western blot analysis (Figure 5A). With CK2-α inhibition, the ER- protein was also assessed as a control protein because ER- is a target protein of CK2-α and is thus expected to be downregulated upon CK2-α inhibition [33]. As expected, ER- protein expression was diminished upon CK2-α downregulation (data not shown). The expression of an siRNA pool for each protein decreased proliferation by more than 60% in the case of siCK2-α, siCCNJ, and siMEGF9. However, siENPEP inhibited cell proliferation by approximately 80% (Figure 5B and 5C). The reduction in proliferation induced by the inhibition of ENPEP, CK2-α, CCNJ, and MEGF9 was accompanied by an increase in G2/M arrest (Figure 5D). In addition, apoptosis analysis was performed upon transfection of each siRNA in MCF7 cells. Repression of each protein did not cause an increase in apoptosis rates compared to scrambled siRNA-expressing control cells (Figure S5A).

To confirm these observations and to eliminate the possibility of off-target effects, we inhibited the genes described above with a different approach, that of shRNA. MCF7 cells were stably transfected with the corresponding shRNAs against each gene and were selected with puromycin for 3 days. A total of four different shRNAs against each gene were used. Therefore, four different cell lines were generated for each gene knockdown (each corresponding to a different shRNA construct). Only those shRNAs that strongly inhibited the target protein were used for further proliferation studies (Figure S5B). The growth curve, cell cycle, and apoptosis profiles were analyzed for each cell line. The results confirmed our previous observations with the siRNA treatments (data not shown).

### ENPEP, CK2-α, CCNJ, and MEGF9 in breast tumors

**Figure 6:**
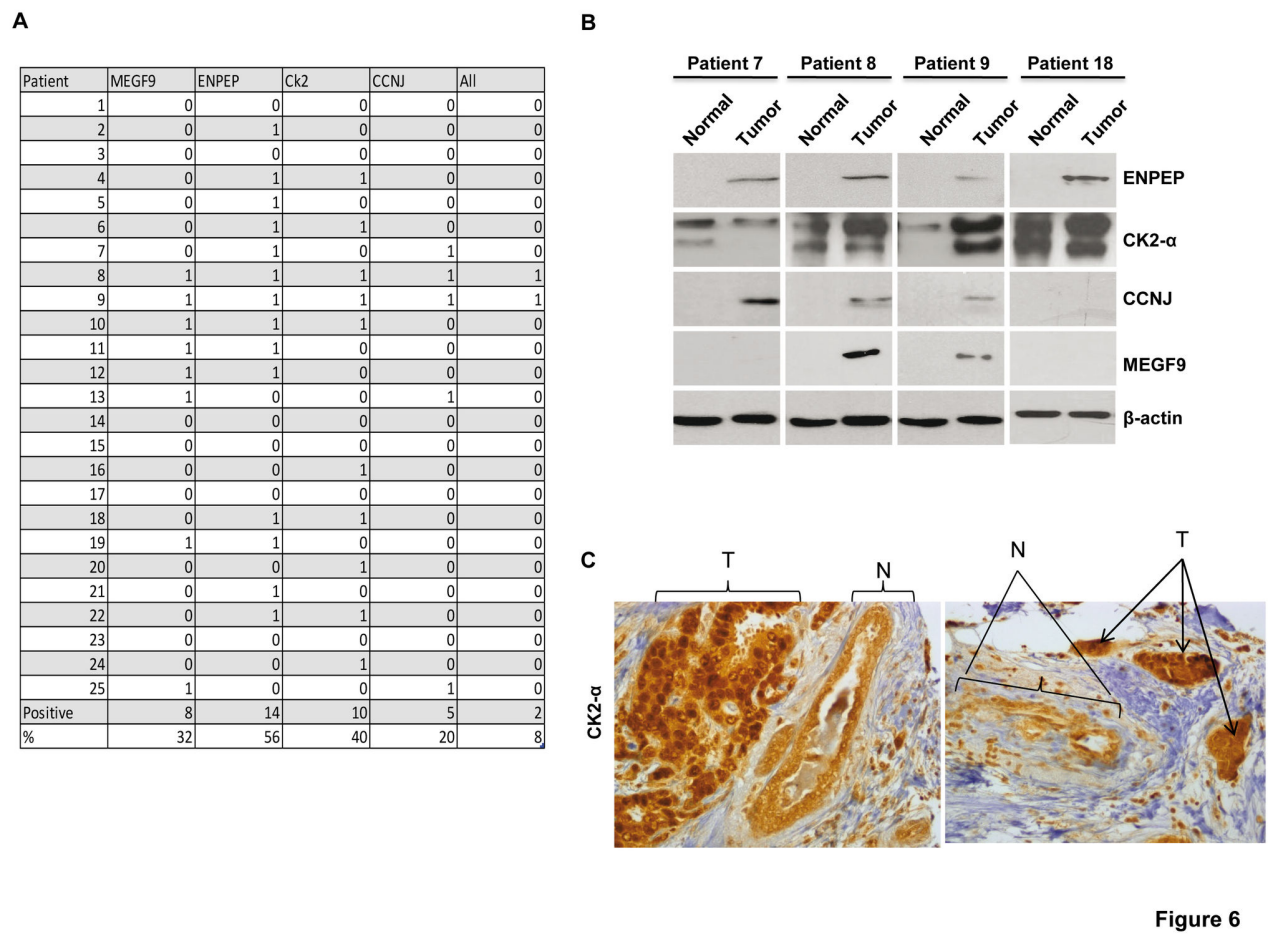
The expression of ENPEP, CK2-α, CCNJ, and MEGF9 in breast tumors. (**A**) Table showing a summary of ENPEP, CK2-α, CCNJ, and MEGF9 expression by western blot in 25 breast tumors. The total percentages of patients that express/overexpress these proteins are shown. 0, lack of expression; 1, positive expression/overexpression. (**B**) Protein expression analyzed by western blot of ENPEP, CK2-α, CCNJ, and MEGF9 in four breast cancer patients is indicated. (**C**) IHC analysis of two patients overexpressing CK2-α. Note the difference in the staining of the tumor (T) in comparison with that of normal (N) breast tissue.

To investigate the importance of ENPEP, CK2-α, CCNJ, and MEGF9 proteins in breast tumors, we studied the expression of each protein by western blot analysis in 25 breast cancer patient samples (series 3; Figure 6A and 6B). In addition, CK2-α was also detected by IHC in the samples from our initial series of 50 patients (series 1).

ENPEP protein was not detected in any patient in normal tissue but was expressed in the corresponding tumor tissues in 56% of patients (Figure 6A). CK2-α was expressed in normal and tumor tissues, but its expression was higher in the tumors, a result that was found on both western blot analysis and IHC (Figure 6B and 6C). In comparison with the levels of normal tissue, CK2-α was overexpressed in the tumor tissue of 56% of the patient samples by IHC and overexpressed in 40% of the patient samples by western blot analysis (Figure 6). CCNJ was expressed in 20% of the breast cancer patient samples and was not detected in the normal breast tissue of any patient (Figure 6A and 6B). Finally, in the 25 frozen biopsies, 32% of the samples exhibited MEGF9 overexpression (Figure 6A and 6B). MEGF9 was not expressed in any patient in normal breast tissue but was detected in the corresponding tumor samples by western blot analysis (Figure 6A and B).

**Figure 7:**
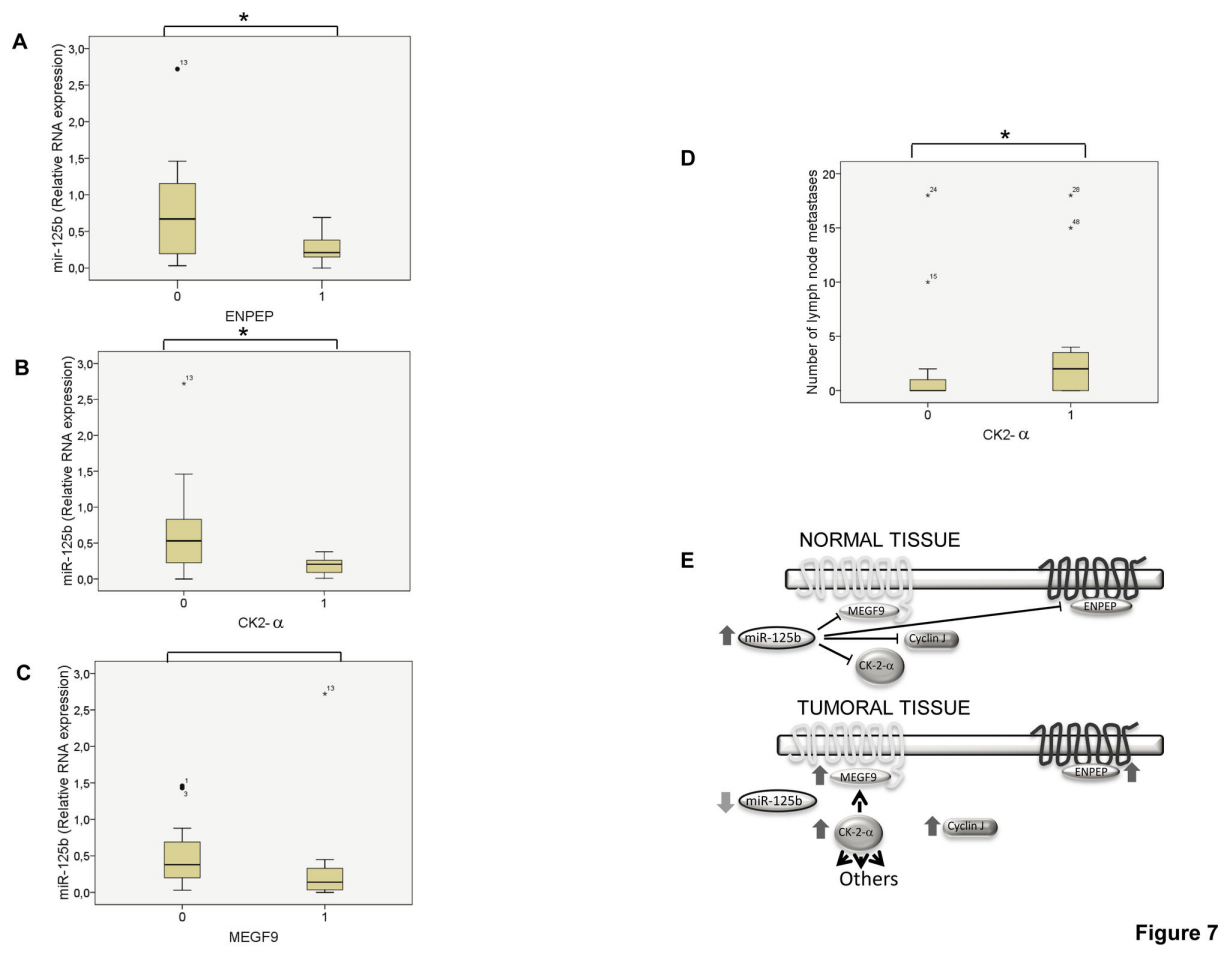
ENPEP, CK2-α, CCNJ, and MEGF9 proteins and their correlation with miR-125b expression in breast cancer patients. (**A**) Correlation between miR-125b expression and ENPEP. (**B**) Correlation between miR-125b expression and CK2-α. (**C**) Correlation between miR-125b expression and MEGF9. (**D**) Correlation between CK2-α expression and the number of lymph node metastases (0, negative CK2-α expression; 1, positive CK2-α expression). (**E**) Schematic representation of how miR-125b might modulate these proteins and their possible interactions. *p < 0.05.

In addition, correlation analysis between miR-125b levels (analyzed with qRT-PCR) and the expression of ENPEP, CK2-α, CCNJ, and MEGF9 protein was studied in 25 breast cancer patient samples (series 3). An inverse correlation between miR-125b and the expression of ENPEP and CK2-α proteins was identified (Figure 7A and 7B). No significant results were detected for MEGF9 and CCNJ, although a clear trend for the MEGF9 protein could be observed (Figure 7C and data not shown).

Furthermore, we investigated whether the expression of ENPEP, CK2-α, CCNJ, and MEGF9 proteins correlated with the pathological characteristics of the patients. Of note, CK2-α expression correlated with the number and presence of lymph node metastases (Figure 7D, and data not shown).

## Discussion

In this study, we present data from genome-wide miRNA expression profiling in different sets of normal and cancerous breast tissues. Our analyses, which involved the most recently identified miRNAs, provide an updated overview of miRNA expression in breast cancer. In the last decade, several studies, performed to establish which miRNAs are aberrantly expressed in breast cancer, have identified key miRNAs in breast cancer progression, as well as miRNA signatures associated with the different molecular types of breast cancer [11,34-36]. In the present work, we identified a total of 35 miRNAs aberrantly expressed in the tumor sample compared with normal breast tissue from the same patient (Figure 1A). We confirmed the downregulation or upregulation of the most dysregulated miRNAs from our study, including potential oncogenic miRNAs (miR-21, miR-96, miR-141, miR-106b, miR-130b, and miR-340) and potential tumor suppressor miRNAs (miR-125b, miR-451, miR-99a, miR-145, miR-100, miR-195, miR-497, miR-376c, miR-486-5p, miR-204, miR-218, miR-381, miR-489, miR-125b-2, miR-299-5p, miR-154, miR-564, and miR-495) reported in previous studies, which further validate our array [11,36-38]. Several of these miRNAs are potential biomarkers for the diagnosis of preinvasive breast lesions [34,39,40]. To our knowledge, our study is the first to propose several miRNAs involved in breast tumorigenesis as potential oncogenic or tumor suppressor miRNAs, including miR-1274a, miR-1274b, miR-1260, miR-329, miR-139-5p, miR-145-3p, miR-1271, miR-139-3p, miR-564, miR-551b, miR-129-3p, and miR-548i. The relevance and importance of these miRNAs should be examined in future studies.

We have also established a profile of 10 significantly dysregulated miRNAs as a fingerprint of breast tumorigenesis. miRNA expression profiling studies with samples from an independent series of patients confirmed all miRNAs contained in the miRNA signature, with the exception of miR-21. The fact that the second series was analyzed at the level of pools (see Materials and Methods) might have masked the important role of miR-21 in breast cancer that was found previously by us and others (Figure 1A and 1B) [23,41]. These results support the proposed list of 10 miRNAs as a reliable fingerprint to assess breast tissue malignancy.

We found that miR-125b is the most significantly downregulated miRNA in breast cancer patients. The prognostic role of miR-125b has been previously suggested in relation with patient survival [18], a parameter that will be analyzed in our series once 5 years have elapsed from the surgery.

miR-125b has been demonstrated to be involved in several diseases, such as psoriasis, Alzheimer’s disease, and cancer. It also modulates some key proteins that are involved in tumorigenesis [13,15,42,43]. However, its role in breast cancer is only now emerging, though downregulation of miR-125b in breast tumors has been described in several reports [11,18,34,44]. Consistent with previous studies [18,30], we corroborated the tumor suppressor role of miR-125b in breast tumorigenesis by demonstrating the following: (1) its strong downregulation in breast tumors, (2) its effect on decreasing the proliferation of mammary cell lines with different degrees of malignancy, and (3) its ability to decrease cellular transformation. We found that the decrease in proliferation observed with miR-125b expression elicits a different response depending on the cell line. Thus, apoptosis is strongly induced in MDA-MB-231 cells, and G2/M cell cycle arrest is induced in HMEC, MCF7, and MDA-MB-231 cells upon miR-125b expression. This finding highlights the functional plasticity of miR-125b according to the cellular context. Importantly, we included HMEC, which are wild-type cells of epithelial origin.

The apoptosis induction observed in MDA-MB-231 cells may be due to alterations associated with the nature of highly tumorigenic cells, in contrast to MCF7 or normal HMEC cells. miR-125b can regulate apoptosis via direct repression of proapoptotic regulators, such as p53, Bak1, and Puma, or antiapoptotic regulators, such as Bcl2, Mcl-1, and Bcl-w, promoting inhibition or induction of apoptosis depending on the cell context [17,29,31,45,46]. Accordingly, we observed downregulation of known miR-125b targets, such as p53, along with their associated partners and targets (e.g., hdm2 or p21^CIP1^) in the cell lines with wild-type p53 (HMEC and MCF7) [29]. However, the phenotypic effect observed in our cells upon miR-125b overexpression could not be due to p53 downregulation itself. Moreover, other proteins reported to be modulated by miR-125b, such as p-ERK, p-AKT, and members of the E2F family, were not affected in our cells [15].

To elucidate the proteins responsible for this effect, we explored other potential oncogenic proteins that could be modulated by miR-125b. Importantly, we found novel miR-125b targets, including ENPEP, CK2-α, CCNJ, and MEGF9. Moreover, the effects of miR-125b on the same target proteins in the three different cell lines (with the exception of CCNJ, which is not expressed in HMEC cells) appear to be a generalized response in mammary cells. Other cell cycle proteins that are not targets of miR-125b were also downregulated, such as CCND1 and CCNB1. This may reflect the cell cycle arrest that is observed in the G2/M phase in the normal cells (HMEC).

The relevance of ENPEP, CK2-α, CCNJ, and MEGF9 proteins in cancer is gradually being revealed. Cell surface peptidases, including ENPEP, play key roles in the regulation of growth, differentiation, and signal transduction of many cellular systems by modulating the activities of the peptide substrates and regulating access to their receptors [47,48]. ENPEP is considered to be an essential and highly specific enzyme, which metabolizes and inactivates bioactive peptides such as angiotensin II (AngII) in the renin-angiotensin system [49]. The putative oncogenic role of ENPEP was demonstrated by Marchio and colleagues [50], who showed that ENPEP regulates blood vessel formation in mice and can serve as a functional vascular target. In addition, ENPEP is expressed in neoplastic lesions of the uterine cervix, and its levels are upregulated as the lesions progress from cervical intraepithelial neoplasms to invasive squamous cell carcinomas [51]. Here, we demonstrated for the first time that ENPEP knockdown has a strong effect on the decreased proliferation of MCF7 cells, which contrasts with the observations from the inhibition of other targets modulated by miR-125b, such as CK2-α, CCNJ, or MEGF9. Moreover, we discovered that ENPEP is overexpressed in a relatively high percentage of breast cancer patients (56%). Accordingly, ENPEP expression is absent in normal cells and is overexpressed in cancer cell lines compared with normal cells of mammary origin (data not shown).

The CK2 protein is a highly conserved serine/threonine kinase. It is ubiquitous in eukaryotic cells, and mainly exists as a tetrameric complex consisting of two catalytic subunits (α, α’) and two regulatory β subunits. CK2-α is a remarkable multifunctional protein kinase with hundreds of substrates, many of which are critically involved in the maintenance of cell viability in normal cells during embryogenesis, and it regulates multiple pathways, including the PI3K/AKT and WNT signaling cascades, NF-κB, and the DNA damage response [33]. CK2-α is considered a prototypical non-oncogene needed for maintaining the transformed phenotype. CK2-α is upregulated in some cancers, including breast cancer, and is involved in modulating proliferation and invasion [52-54]. In the present study, we have shown that CK2-α knockdown decreases the proliferation of MCF7 cells by inducing G2/M cell cycle arrest. This finding is consistent with a previous report that showed that treatment of breast cancer cells with a chemical inhibitor of CK2-α induced G2/M cell cycle arrest [55]. Moreover, we have found that CK2-α protein is expressed in 40%–56% of breast cancer patients.

MEGF9 is a transmembrane protein that is strongly expressed in the nervous system and is regulated during development [56]. A study has suggested its possible association with cancer, demonstrating a link between a decrease in MEGF9 expression and the aggressiveness of soft tissue tumors [57]. Our results are not, however, consistent with the results of Cunha et al. [57], possibly because that study focused on soft tissue tumors as opposed to the solid tumors that we analyzed here. In addition, the results of Cunha et al. [57] were based solely upon RNA expression data, which requires further confirmation by protein analysis. For the first time, our current data show that MEGF9 is capable of regulating cell proliferation because MEGF9 knockdown—by different strategies—significantly decreased the proliferation of MCF7 cells. Moreover, we found that MEGF9 protein is expressed in 32% of tumors, a finding that could have therapeutic implications in breast cancer patients as this protein is a membrane receptor whose blockage might alter the proliferative signal.

Finally, CCNJ, a protein that controls cell mitosis, is a distinctive cyclin characterized by its exclusive maternal expression pattern, which suggests that it may regulate oogenesis and embryogenesis [58,59]. CCNJ is not a ubiquitous cyclin because it is not expressed in some tissues or in certain cancer cell lines with origins other than mammary tissue (www.proteinatlas.com and data not shown). Moreover, its peculiar expression in the cytoplasm also differentiates it from the other cyclins (www.proteinatlas.com). CCNJ is a putative oncogene because is embedded in a cluster of genes found dysregulated in pediatric high-risk B-precursor acute lymphoblastic leukemia [60]. The fact that CCNJ is not expressed in normal HMEC cells but is expressed in certain cancer cells (MCF7 and MDA-MB-231) and that CCNJ silencing decreased the proliferation of MCF7 cells by inducing G2/M cell cycle arrest is consistent with the proliferative role of CCNJ suggested by other studies in *Drosophila* and prostate cancer cells [59,61]. Importantly, CCNJ expression is present in 20% of patients with breast cancer, exclusively in the malignant tissues. Therefore, we suggest a potential oncogenic role of CCNJ in breast tumorigenesis.

The aim of analyzing ENPEP, CK2-α, CCNJ, and MEGF9 expression in breast tumors was to clarify their association with miR-125b. Remarkably, miR-125b is inversely correlated with ENPEP and CK2-α expression in breast tumors. miR-125b also trends towards a correlation with MEGF9, though this association needs to be confirmed by further studies. Our results establish these proteins as relevant *in vivo* miR-125b targets with potential important clinical implications in breast tumorigenesis. Specifically, CK2-α overexpression is associated with the presence and number of lymph node metastases. This fact underscores the usefulness of CK2-α as a prognostic marker for breast tumors, as previously suggested for colon and breast cancers [62,63]. Regarding the lack of an association between miR-125b and CCNJ expression, it is tempting to hypothesize that miR-125b may correlate with CCNJ levels in certain tumorigenic cells (e.g., cancer stem cells). Several reports describe a stemness role for miR-125b and CCNJ, which would explain why its expression is associated with stem cells but not mature differentiated cells [64-66]. Apart from the overexpression of ENPEP, CK2-α, CCNJ, and MEGF9 in certain breast cancer patients, the potential oncogenic roles of these proteins is supported by the fact that their downregulation causes significant inhibition of cell proliferation. Our data suggest that in some percentage of patients with breast cancer tumors, miR-125b downregulation consistently occurs; this involves a repression of the genes that code for ENPEP and CK2-α, and potentially MEGF9 and CCNJ (Figure 7E). Therefore, miR-125b modulates the different molecular pathways associated with these proteins. Moreover, dysregulation of these oncogenic proteins might contribute to a great extent to the tumorigenic phenotype, which is explained, at least in part, by miR-125b downregulation. The pathways in which these proteins are involved appear to play important roles in cancer cells derived from breast tissues, regardless of their ER-α or p53 status (which differ among the cell lines studied). Expanding our knowledge of the pathways that are used by these proteins to decrease proliferation *in vivo* (e.g., angiogenesis, cell cycle, or receptor signaling) would open new avenues for the treatment of breast cancer.

## Supporting Information

Figure S1 **miR-125b expression in HMEC and MCF-7 cells.** (A) Expression of the cell cycle-related proteins p53, p21^CIP1^, hdm2, and p16^INK4A^ in HMEC cells expressing miRVec-GFP or miRVec-125b (left panel). Expression of p-AKT, p-MAPK, E2F1, and E2F2 in miRVec-GFP- and miRVec-125b-expressing HMEC cells (right panel). (B) 3T3 protocol in miRVec-GFP- and miRVec-125b-expressing HMEC cells. (C) miRVec-GFP- and miRVec-125b-transduced HMEC and MCF7 cells were stained with β-galactosidase to detect the presence of senescent cells. A senescence-induced control is included for each cell line. OHT, tamoxifen. **p < 0.01, ***p < 0.001.
(TIF)

Figure S2 **miR-125b expression and apoptosis analysis.** (A) Western blot analysis of PARP in the different cell lines used in this study (HMEC, MCF7, and MDA-MB-231). (B) Apoptosis rates analyzed by FACS of miRVec-GFP- and miRVec-125b-transduced HMEC and MCF7 cells. The percentages of cells in early (lower right panel) and late (upper right panel) apoptosis are indicated for each cell line.
(TIF)

Figure S3 **Dose-response of anti-125b in HEK293T cells.** Inhibitory effects of different anti-125b concentrations (10–100 nM) on the endogenous levels of miR-125b, as assessed by qRT-PCR. Note the progressive decrease in miR-125b expression with the increasing levels of anti-125b.
(TIF)

Figure S4 **Different mutants of the 3’-UTRs of ENPEP, CK2-α, CCNJ, and MEGF9.** Alignment of the 3’-UTRs of ENPEP, CK2-α, CCNJ, and MEGF9 mRNAs and the predicted conserved miR-125 binding sites at the indicated positions. The point mutations that were introduced in the 3’-UTR of each mutant gene construct are indicated in bold. The seed sequence is indicated in yellow.
(TIF)

Figure S5 **Knockdown of ENPEP, CK2-α, CCNJ, and MEGF9.** (A) Apoptosis detection by FACS upon transient transfection of the indicated siRNAs and their controls. (B) Western blot analysis of CCNJ and MEGF9 in scrambled negative control MCF7 cells, shCCNJ-transduced MCF7 cells (4 different shRNAs), and shMEGF9-transduced MCF7 cells (4 different shRNAs). shCCNJ (No. 2) and shMEGF9 (No. 1) were selected for protein studies, as well as cell cycle, apoptosis, and growth curve studies.
(TIF)

Table S1 **Description of primers used in this article.**
(XLS)

Table S2 **Row data from miRNA arrays.** The results from the miRNA arrays for all miRNAs are shown for each patient (T, tumor). Pools of normal tissue (N) are also indicated (pool A, pool B, and pool C). Fold-change (FC) values are shown for each miRNA, as well as the p value and p-adjusted value, as described in the Materials and Methods. A logarithmic scale for the T/N ratio is shown.
(XLS)

Table S3 **Differentially expressed miRNAs in an independent series of patients.** Pools of tumor and normal tissue were analyzed in duplicate by different array platforms, as described in the Materials and Methods. Green indicates the miRNAs that were significantly downregulated in the tumor in relation with the normal tissue. Orange indicates that the miRNAs were upregulated.
(XLS)
