# Supplementary figures and images for: miR-125b Acts as a Tumor Suppressor in Breast Tumorigenesis via Its Novel Direct Targets ENPEP, CK2-α, CCNJ, and MEGF9
Source: PLoS One. 2013 Oct 3;8(10):e76247. doi: 10.1371/journal.pone.0076247 (PMC3789742; doi:10.1371/journal.pone.0076247)

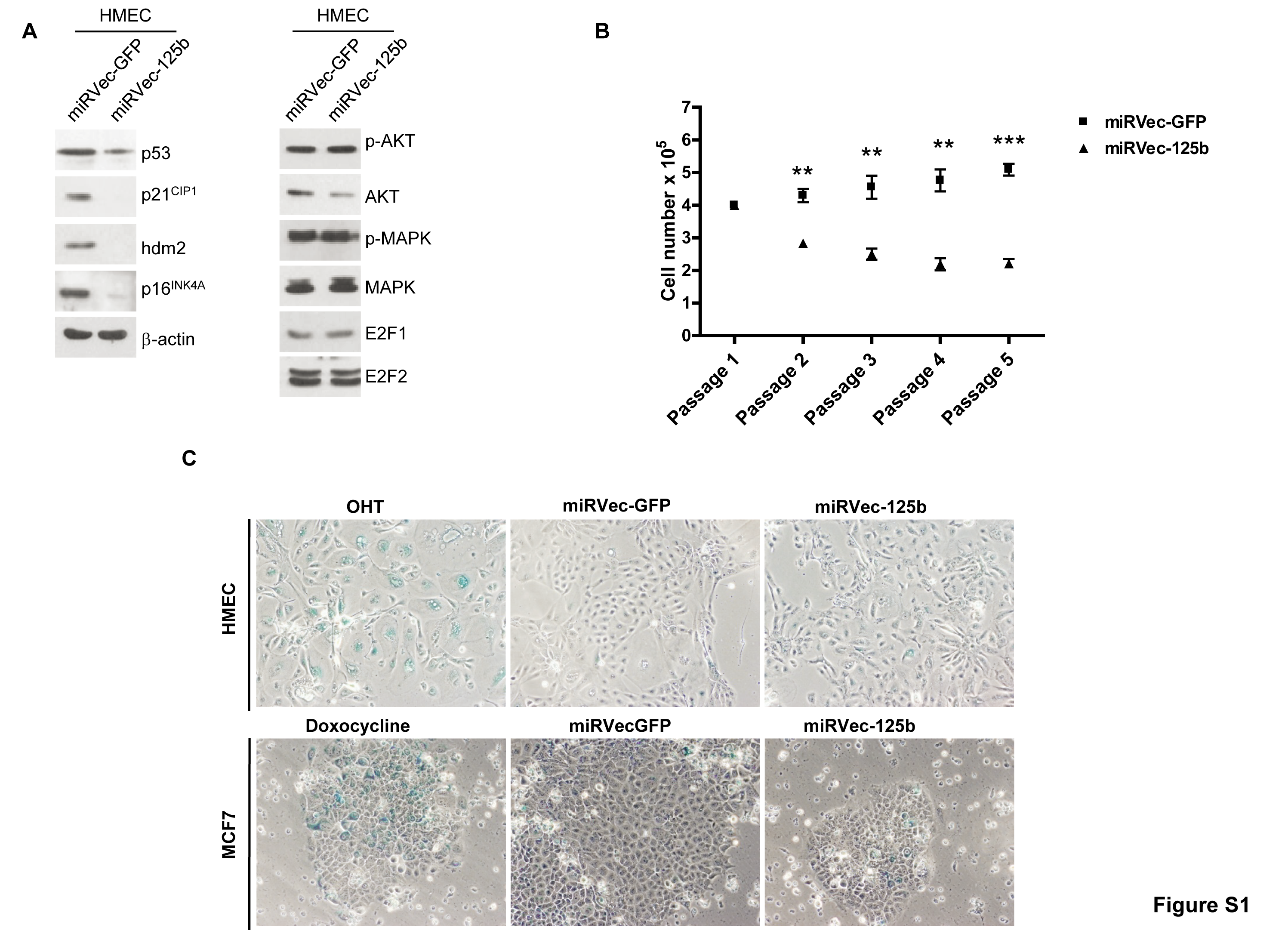

Supplement: Figure S1 — miR-125b expression in HMEC and MCF-7 cells. (A) Expression of the cell cycle-related proteins p53, p21CIP1, hdm2, and p16INK4A in HMEC cells expressing miRVec-GFP or miRVec-125b (left panel). Expression of p-AKT, p-MAPK, E2F1, and E2F2 in miRVec-GFP- and miRVec-125b-expressing HMEC cells (right panel). (B) 3T3 protocol in miRVec-GFP- and miRVec-125b-expressing HMEC cells. (C) miRVec-GFP- and miRVec-125b-transduced HMEC and MCF7 cells were stained with β-galactosidase to detect the presence of senescent cells. A senescence-induced control is included for each cell line. OHT, tamoxifen. **p < 0.01, ***p < 0.001. (TIF) [file pone.0076247.s001.tif]

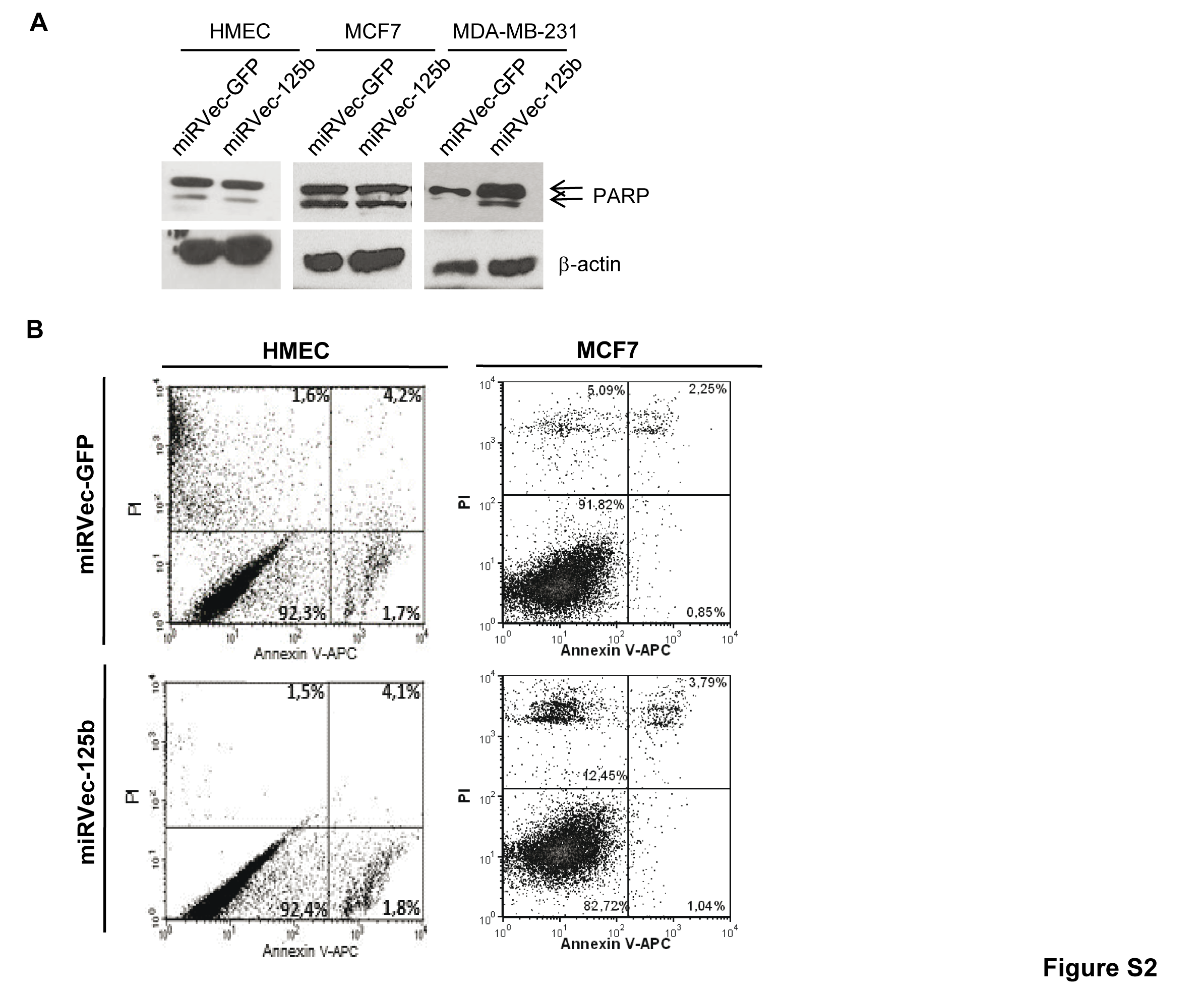

Supplement: Figure S2 — miR-125b expression and apoptosis analysis. (A) Western blot analysis of PARP in the different cell lines used in this study (HMEC, MCF7, and MDA-MB-231). (B) Apoptosis rates analyzed by FACS of miRVec-GFP- and miRVec-125b-transduced HMEC and MCF7 cells. The percentages of cells in early (lower right panel) and late (upper right panel) apoptosis are indicated for each cell line. (TIF) [file pone.0076247.s002.tif]

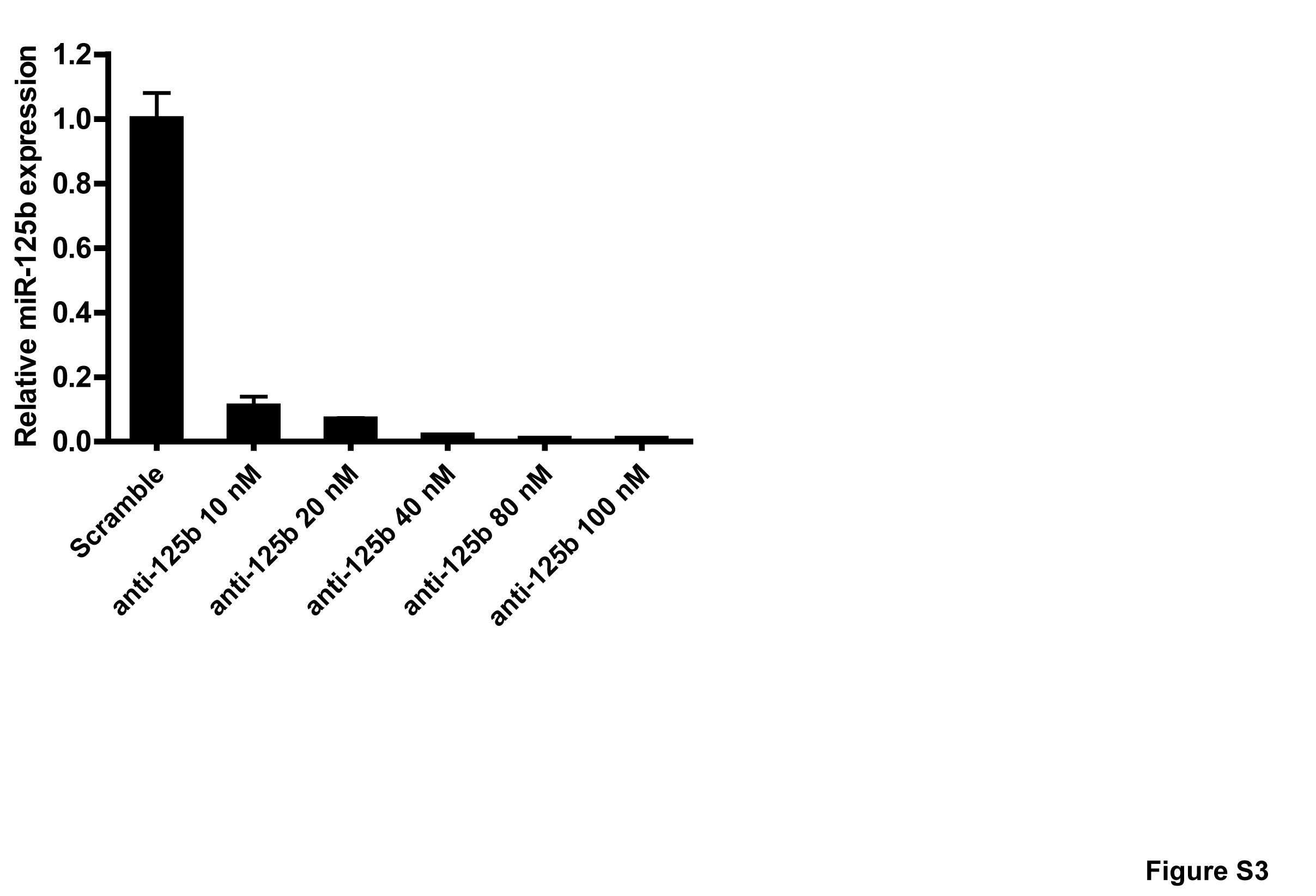

Supplement: Figure S3 — Dose-response of anti-125b in HEK293T cells. Inhibitory effects of different anti-125b concentrations (10–100 nM) on the endogenous levels of miR-125b, as assessed by qRT-PCR. Note the progressive decrease in miR-125b expression with the increasing levels of anti-125b. (TIF) [file pone.0076247.s003.tif]

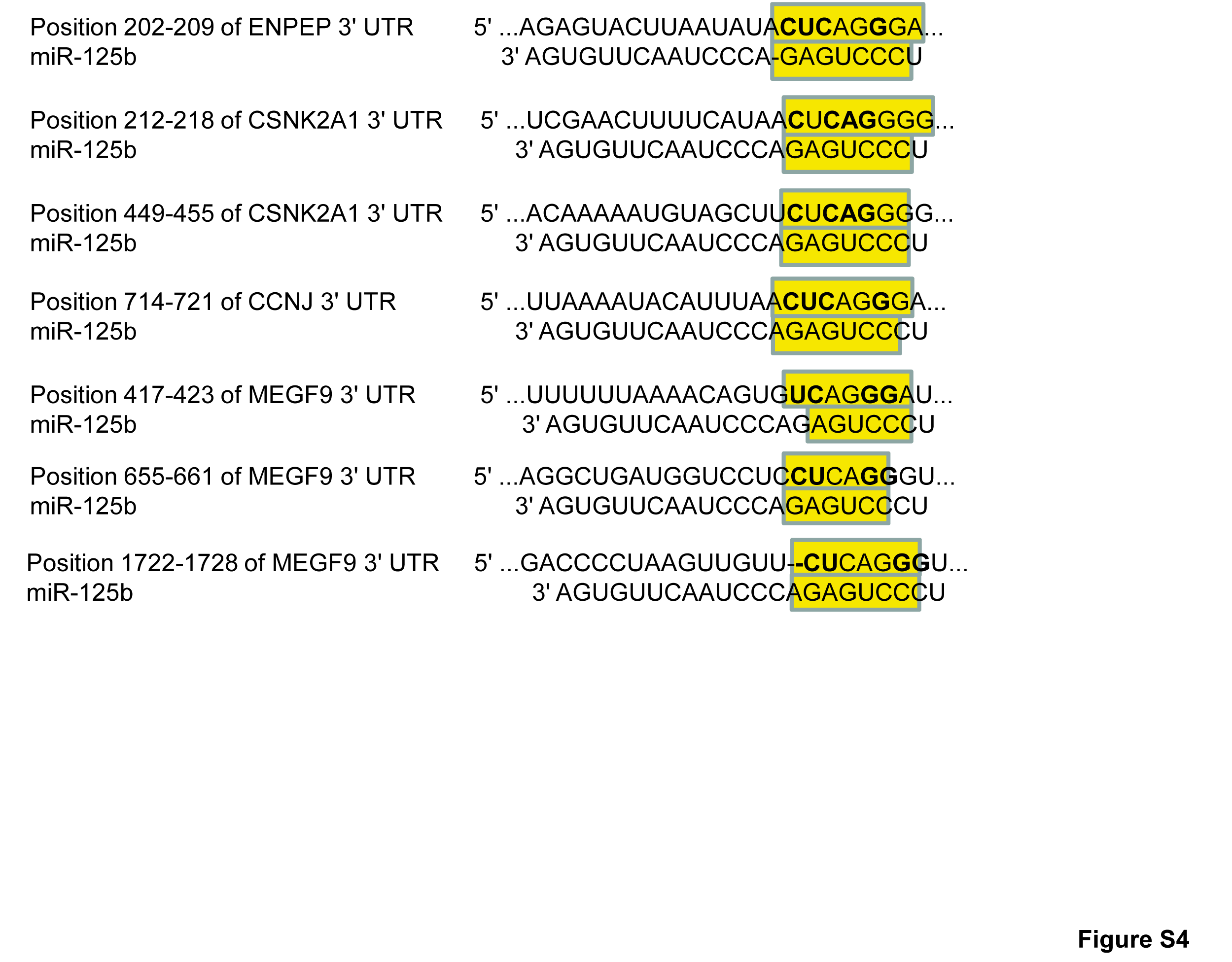

Supplement: Figure S4 — Different mutants of the 3’-UTRs of ENPEP, CK2-α, CCNJ, and MEGF9. Alignment of the 3’-UTRs of ENPEP, CK2-α, CCNJ, and MEGF9 mRNAs and the predicted conserved miR-125 binding sites at the indicated positions. The point mutations that were introduced in the 3’-UTR of each mutant gene construct are indicated in bold. The seed sequence is indicated in yellow. (TIF) [file pone.0076247.s004.tif]

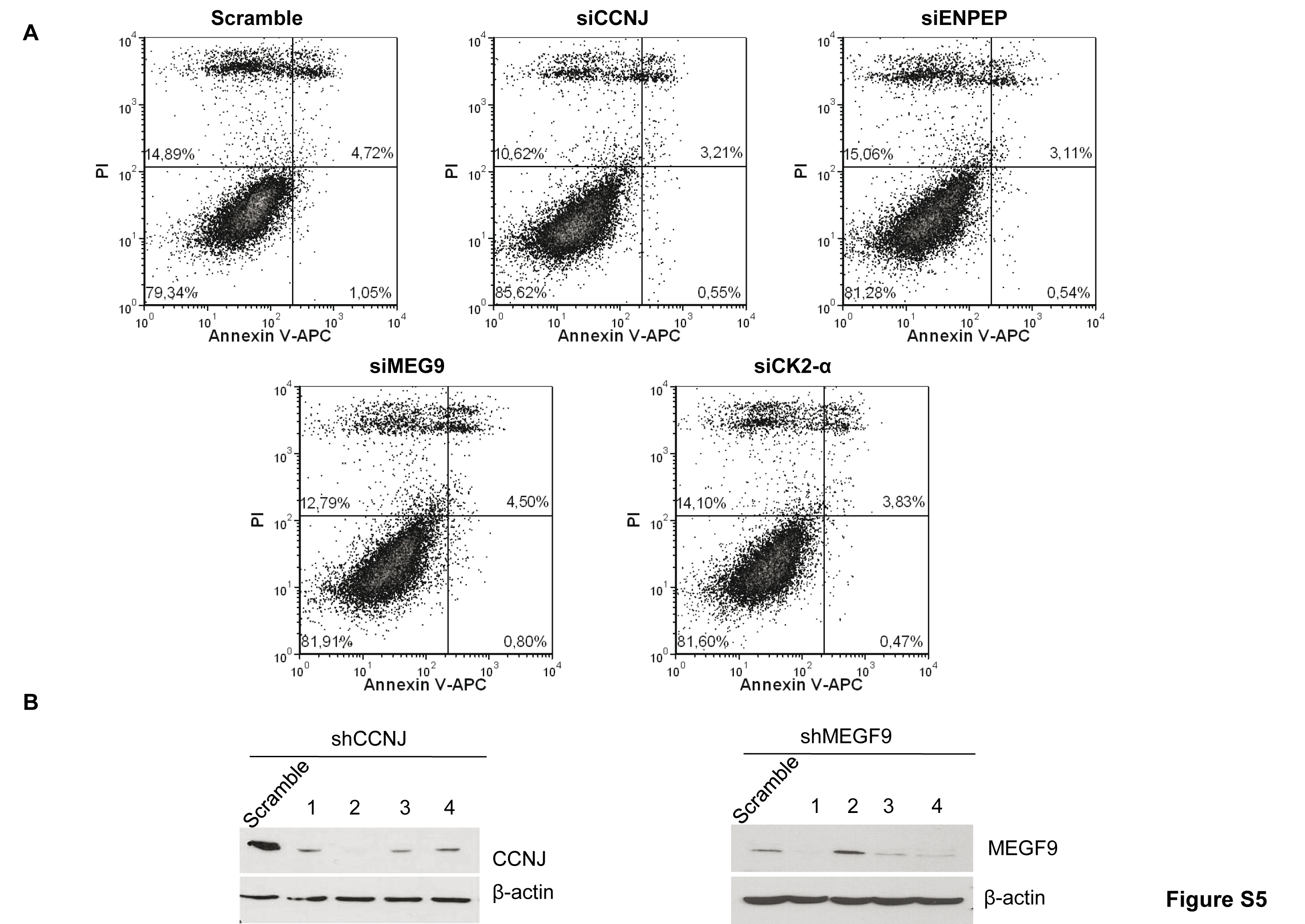

Supplement: Figure S5 — Knockdown of ENPEP, CK2-α, CCNJ, and MEGF9. (A) Apoptosis detection by FACS upon transient transfection of the indicated siRNAs and their controls. (B) Western blot analysis of CCNJ and MEGF9 in scrambled negative control MCF7 cells, shCCNJ-transduced MCF7 cells (4 different shRNAs), and shMEGF9-transduced MCF7 cells (4 different shRNAs). shCCNJ (No. 2) and shMEGF9 (No. 1) were selected for protein studies, as well as cell cycle, apoptosis, and growth curve studies. (TIF) [file pone.0076247.s005.tif]
